# Modeling of inflicted head injury by shaking trauma in children: what can we learn? Part II: A systematic review of mathematical and physical models

**DOI:** 10.1007/s12024-019-00093-7

**Published:** 2019-02-20

**Authors:** Jan Peter van Zandwijk, Marloes E. M. Vester, Rob A. Bilo, Rick R. van Rijn, Arjo J. Loeve

**Affiliations:** 1 Division of Digital and Biometric Traces, Netherlands Forensic Institute, Laan van Ypenburg 6, 2497 GB The Hague, the Netherlands; 2 Department of Radiology and Nuclear Medicine, Academic Medical Center Amsterdam, Meibergdreef 9, 1105AZ, Amsterdam, the Netherlands; 3 Specialist Services and Expertise Division, Netherlands Forensic Institute, Laan van Ypenburg 6, 2497 GB The Hague, the Netherlands; 4 Department of BioMechanical Engineering, Faculty of Mechanical, Maritime & Materials Engineering, Delft University of Technology, Mekelweg 2, 2628 CD Delft, the Netherlands

**Keywords:** Closed head injuries, child abuse, forensic pathology, rigid body models, finite element models

## Abstract

Various types of complex biomechanical models have been published in the literature to better understand processes related to inflicted head injury by shaking trauma (IHI-ST) in infants. In this systematic review, a comprehensive overview of these models is provided. A systematic review was performed in MEDLINE and Scopus for articles using physical (e.g. dolls) and mathematical (e.g. computer simulations) biomechanical models for IHI-ST. After deduplication, the studies were independently screened by two researchers using PRISMA methodology and data extracted from the papers is represented in a “7-steps description”, addressing the different processes occurring during IHI-ST. Eleven papers on physical models and 23 papers on mathematical models were included after the selection process. In both categories, some models focus on describing gross head kinematics during IHI-ST events, while others address the behavior of internal head- and eye structures in various levels of detail. In virtually all physical and mathematical models analyzed, injury thresholds are derived from scaled non-infant data. Studies focusing on head kinematics often use injury thresholds derived from impact studies. It remains unclear to what extent these thresholds reflect the failure thresholds of infant biological material. Future research should therefore focus on investigating failure thresholds of infant biological material as well as on possible alternative injury mechanism and alternative injury criteria for IHI-ST.

**Electronic supplementary material:**

The online version of this article (10.1007/s12024-019-00093-7) contains supplementary material, which is available to authorized users.

## Introduction

As described in part one of this review [1], inflicted head injury by shaking trauma in infants (IHI-ST) is a subject which has over the years given rise to much discussion in the scientific literature. While part one focuses on reviewing the literature on animal models for IHI-ST, in this paper a literature review is presented on mathematical and physical models that have been developed to understand IHI-ST.

In many fields of biomechanics, including studies investigating IHI-ST, physical and mathematical models are used to answer questions in cases where direct experimentation with animals or children is impossible for practical and ethical reasons. In physical modeling of IHI-ST, a mechanical model (like a doll) of an infant is constructed and subjected to events that are suspected to lead to IHI, such as violent shaking. These physical models, which may be either commercially available test-dolls or proprietary ones, are equipped with measuring devices collecting kinematic or dynamic data from the surrogate. Based on these data, conclusions are then inferred concerning IHI-ST.

In mathematical modeling for IHI-ST, part or all of the infant’s anatomy is represented by sets of mathematical equations to make a simulation model of IHI-ST events. These mathematical models include rigid body models (RBMs) and finite element models (FEMs). **RBMs** for IHI-ST generally focus on simulating gross body kinematics by representing the body of a young child as a set of rigid segments, linked together with hinges of various properties. Such a model can be made to move by, for example, applying external forces to the modelled segments. Output of a rigid body model can include the kinematics of the movement (such as angular- and linear accelerations and velocities of the child’s head). **FEMs** for IHI-ST aim to study how the skull, neck, eyes, and internal structures inside the head of an infant are loaded, and move and deform as a consequence of motions and accelerations applied to the infant. In order to do so, a detailed representation of these structures is created by subdividing these into a large number of small blocks, called ‘elements’. Each element is assigned specific elastic or viscoelastic properties, expressing differences in material properties between various internal structures. The output of a FEM may include deformations or relative displacements of internal structures, as well as internal stresses in the simulated structures, under the applied loading conditions.

One of the major difficulties when studying the literature on physical and mathematical IHI-ST models, is the wide variety of modeling approaches with different strengths and limitations. This diversity makes it hard to compare models with each other, assess the conditions under which these models are applicable in practice, and value their sometimes contradictory conclusions. Besides this, many of these studies are rather technical, which can make them difficult to understand for practitioners working in the field of IHI-ST who are not trained in biomechanics. Therefore, this paper presents a systematic review of the literature on physical and mathematical models for IHI-ST in order to:

1. Systematically organize and represent information of the current state of affairs in mathematical and physical IHI-ST modeling studies to make these accessible to legal and medical practitioners and to make it easier to compare models with each other.
2. Gain a better understanding of what we can learn from these studies about factors likely to affect IHI-ST.
3. Identify gaps in the literature as a direction for future studies.

In order to aid non-technical readers to fully grasp the presented concepts, appendix A offers a basic explanation of some important concepts in biomechanics that will be used frequently throughout this review.

## Methods

### Database search

**Fig. 1:**
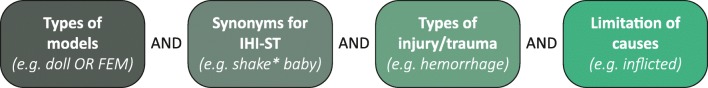
Basic structure of the search queries applied in Pubmed and Scopus

A systematic literature search was performed in PubMed [www.ncbi.nlm.nih.gov/pubmed/] and Scopus [www.scopus.com] for articles up to January 1st, 2017. For each database, search queries were constructed to identify IHI-ST related studies using animal, mathematical or physical models, or any combination thereof (Fig. 1). Search queries used are available as online supplementary material. Only articles written in English, German, French, and Dutch were searched for. The search results from both databases were combined and de-duplicated.

### Article selection

Relevant articles were selected from the de-duplicated results using the PRISMA methodology [www.prisma-statement.org]. Papers were screened for their relevance on understanding or explanation of (aspects of) IHI-ST using a physical or mathematical model based on title and abstract. Finally, papers were screened on their full texts using a pre-designed evidence assessment sheet and included if a physical or mathematical model was described and multiple cycles of acceleration and deceleration were applied. Each step in the selection process was independently performed by authors AJL and JPvZ. In cases where these two authors disagreed about including or excluding a paper from the review, a consensus was sought. If no consensus was reached, the article was included.

Backward snowballing was performed in which the reference lists of the included articles were screened to find additional relevant articles. These resulting extra articles were put through the same article selection process as the articles found through the database searches.

### Data extraction

Authors AJL and JPvZ independently extracted data from the full texts of all selected articles using a pre-designed data extraction sheet. Extracted information included baseline information, model description (e.g. modeled entities, material properties), model inputs, response and injury criteria used (e.g. injury thresholds), and main study outcomes.

### Data structuring

**Fig. 2:**
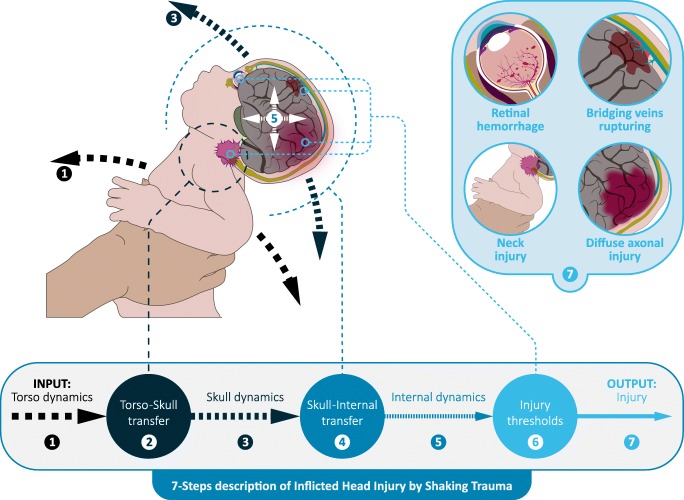
The seven steps describing the mechanics of inflicted head injury by shaking trauma in infants. The odd steps can be considered as system states and the even steps can be considered as transfer functions that describe how, for example, motion of one part of the anatomy is transformed into motion of another part or into injury

In order to systematically organize and compare data extracted from the papers, it was decided to subdivide the event of IHI-ST into 7 consecutive steps (Fig. 2). In this “7-steps description”, the infant is viewed as a system consisting of interconnected moving anatomical elements with various geometries and material properties. Steps 1, 3, 5, and 7 can be considered as system states of the modeled infant and Steps 2, 4, and 6 describe how dynamics of one part affect the behavior of another part: so-called transfer functions.

In the “7-step description”, an IHI-ST event is initiated by a shaking motion exerted onto the infant’s torso, the torso dynamics, which is the input of Step 1. That motion is transferred by the neck to the head in Step 2, which results in the skull dynamics of Step 3. In turn, these skull dynamics determine in Step 4 how everything inside the skull moves and deforms, resulting in the internal dynamics in Step 5. Internal dynamics include loading and deformation of the anatomical elements inside the skull, such as pressure acting on the eyes or strains acting on the bridging veins. The dynamics of internal anatomical elements might lead to damage of these elements if their threshold for material damage is exceeded in Step 6, which then gives rise to the injury in Step 7.

## Results

### Search results

**Fig. 3:**
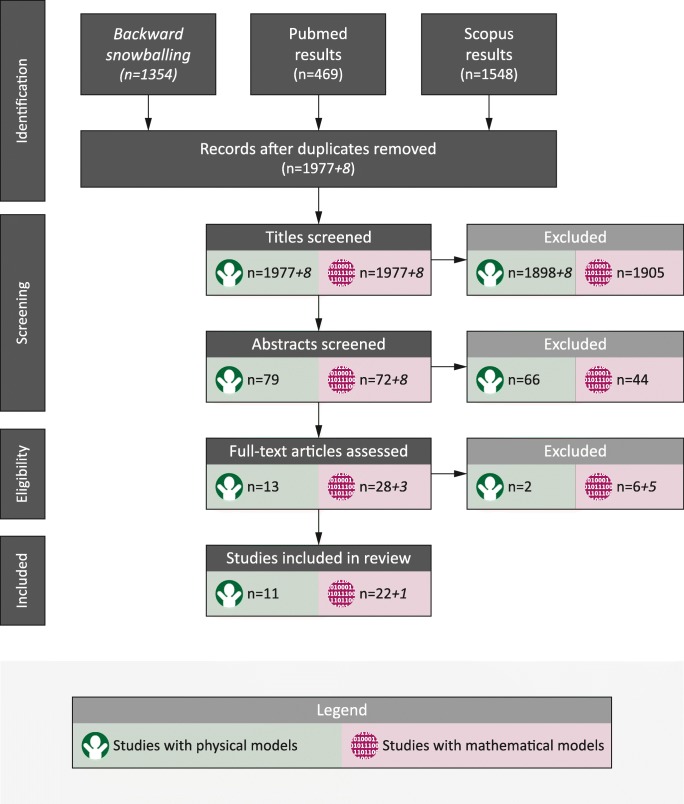
PRISMA flow chart of the conducted literature search and article selection process for papers describing physical and mathematical models for IHI-ST. The numbers printed in italic type with a “+” sign are the numbers of additional articles identified by backward snowballing

After database search and subsequent screening, 11 papers describing physical models and 22 papers describing mathematical models for IHI-ST were included (Fig. 3) in the study. 1354 references citing the 33 included papers were identified. After de-duplication, 8 potentially relevant new papers were selected based on title. After further screening on abstract and full-text, 1 paper on mathematical modeling of IHI-ST was added to the final selection.

### Data extraction results

The complete data extraction sheet is available online as supplementary material. As a general overview, Tables 1 and 2 show which steps of the “7-steps description” for each of the included papers from Fig. 2 it addresses. In general, mechanical models and RBMs tend to focus on the first three steps, and FEMs are more focused on the last 5 steps (Tables 1 and 2).

**Table 1:** Overview of which steps of the 7-steps description of IHI-ST mechanics are addressed in the included papers on physical models. See Fig. 2 for terminology. An entry marked with a bullet indicates that the corresponding phase is explicitly included in the model described in the corresponding paper. In the case of 7. Injury, a bullet indicates that the paper states that injury might occur on the basis of their model

Reference	Publication year	1. Torso Dynamics	2. Torso-skull transfer	3. Skull dynamics	4. Skull internal transfer	5. Internal dynamics	6. Injury thresholds	7. Injury
Duhaime et al. [2]	1987	●	●	●			●	
Jenny et al. [3]	2002	●	●	●				
Cory & Jones [4]	2003	●	●	●			●	●
Prange et al. [5]	2003	●	●	●			●	
Cheng et al. [6]	2010			●	●	●		
Lloyd et al. [7]	2011	●	●	●			●	
Cirovic et al. [8]	2012	●	●	●	●	●		
Koizumi et al. [9]	2013	●	●	●	●	●	●	●
Yamazaki et al. [10]	2014	●	●	●	●	●	●	
Miyazaki [11]	2015	●	●	●	●	●	●	●
Tomlinson & Taylor [12]	2015			●	●	●	●	
Jenny et al.[13]	2017	●	●	●

**Table 2:** Overview of which steps of the 7-steps description of IHI-ST mechanics are addressed in the included papers on mathematical models. See Fig. 2 for terminology. An entry marked with a bullet indicates that the corresponding phase is explicitly included in the model described in the corresponding paper. In case of 7. Injury, a bullet indicates that the paper states that injury might occur on the basis of their model. Margulies et al. [34] is a letter to the editor, commenting on results by Bandak [33]

Reference	Publication year	1. Torso Dynamics	2. Torso-skull transfer	3. Skull dynamics	4. Skull-internal transfer	5. Internal dynamics	6. Injury thresholds	7. Injury
Rigid body models								
Wolfson et al. [14]	2005	●	●	●			●	
Bondy et al. [15]	2014	●	●	●			●	
Jones et al. [16]	2014	●	●	●			●	
Lintern et al.[17]	2015	●	●	●				
Finite Element Models								
Morison [18]	2002			●	●	●	●	●
Cirovic et al. [19]	2005					●		
Roth et al. [20]	2007			●	●	●	●	●
Cheng et al. [21]	2008			●	●	●		
Raul et al. [22]	2008			●	●	●		
Hans et al. [23]	2009		●	●	●	●	●	●
Couper & Albermani [24]	2008			●	●	●		
Couper & Albermani [25]	2008			●	●	●		
Batterbee et al. [26]	2009			●	●	●		
Rangarajan et al. [27]	2009				●	●		
Cheng et al. [6]	2010			●	●	●		
Couper & Albermani [28]	2010			●	●	●	●	●
Batterbee et al. [29]	2011			●	●	●		
Ponce & Ponce [30]	2011		●	●	●	●	●	
Coats et al. [31]	2012				●	●	●	
Yoshida et al. [32]	2014					●		
Nadarasa et al. [33]	2015					●		●
Other								
Bandak [34]	2005		●	●			●	
Margulies et al. [35]	2006							

### Outcomes per step of the 7-steps description

#### Step 1. Torso dynamics

##### Physical models

In the majority of papers describing physical models, a doll was shaken by one or more volunteers. Instructions given to the volunteers vary, but often come down to: “shake as violently as possible”. In some studies, an explicit distinction was made between shaking in the sagittal plane while keeping the infant more or less upright and “gravity assisted shaking” where shaking is accompanied by a forceful up- and downward acceleration of the infant (i.e. Cory & Jones [4] and Lloyd et al. [7]). Accelerations at both the hands of the volunteers and at the head of the doll were only measured directly in Yamazaki et al. [10], thus taking into account Steps 1 to 3 in Fig. 2. Typically, shaking frequencies and durations attained during human shaking experiments were 2-5 Hz for 3-5 seconds. In Cheng et al. [6] and Koizumi et al. [9] machine-based shaking was applied, using actuators to apply shaking frequencies of about 3.5 Hz and 1.5 – 3.5 Hz, respectively.

##### Mathematical models

In RBM studies on IHI-ST, researchers use experimental data on torso kinematics to drive their models, either by using these as a kinematic constraint (e.g. prescribing the movement of the modelled torso) or as an external force acting on the torso. Lintern et al. [17] experimentally measured torso motion while shaking a lamb is used as input to their lamb RBM. In Jones et al. [16] a sinusoidal shake of 3Hz and amplitude of 65mm is applied to their model, based on human shaking data from Cory & Jones [4]. Wolfson et al. [14] used experimental data on linear torso acceleration from shaking a doll as input to their model. Bondy et al. [15] used the same experimental data to drive their model.

FEMs generally model only part of the body (typically the head or eye and their internal structures) during IHI-ST events. Therefore, torso dynamics is not part of the modeling process.

#### Step 2. Torso-skull transfer

##### Physical models

In doll studies, head kinematics is strongly dependent on the coupling between the torso and the head: the neck. In studies that included effects of neck properties on the torso-skull transfer, either proprietary test dolls or commercial crash-test dummies were used for experimentation. In studies using proprietary dolls [2–5, 8, 10, 13] different neck types were tested, ranging from elastic tubing to frictionless hinge joints. In these studies, biofidelity of the neck models was either not discussed or stated to be unknown. Prange et al. [5] argued that their hinge type neck might be appropriate for new-borns, who cannot support the weight of their heads, and that these experiments provided an upper bound for head kinematics during IHI-ST. Jenny et al. [3, 13] are the only ones providing quantitative data of the neck stiffness of their proprietary doll. Overall, there seems to be a consensus that quantitative data on biomechanical properties of the infant’s neck is lacking. In studies using commercial crash-test dummies [7–9, 11], biofidelity of the dolls was not discussed in detail.

##### Mathematical models

Two RBM studies investigated the role of passive neck stiffness on skull dynamics [14, 16]. In Jones et al. [16] it is reported that peak linear accelerations at the vertex during simulated IHI-ST events can increase sevenfold depending on the necks stiffness. Wolfson et al. [14] varied the neck stiffness profile in their model and reported that resulting head dynamics for all shaking simulations and all tested neck stiffnesses were below the injury thresholds for head injury against which the results were compared (see Step 6. Injury thresholds). Only when end-point constraints were encountered in the simulations, causing the head to impact on the chest and back during shaking, were head dynamics found to exceed injury thresholds. Bandak [34] calculated neck distraction forces from angular velocities and accelerations taken from literature. On the basis of these calculations, it was argued that the forces required to sustain angular velocities and angular accelerations during IHI-ST exceed the limits of structural failure of the infant’s spine, implying that IHI-ST caused by shaking alone would be unlikely to exist without neck injuries. However, Margulies et al. [35] recalculated neck distraction forces using data from Bandak [34] and criticized the forces reported in [34] as being at least ten times too large.

As in Step 1, none of the selected FEM studies addresses torso-head transfer: shaking inputs were applied directly to the head or eye.

#### Step 3. Skull dynamics

##### Physical models

In doll studies, head dynamics and kinematics are often measured using accelerometers embedded on different locations in the doll’s head. In many of these studies, overall head kinematics are the main results reported (Table 3). Three studies suggested that the largest angular accelerations in IHI-ST occurred during chin-to-chest or occiput-to-back contact [4, 7, 13].

**Table 3:** Values of kinematic parameters as reported in papers on mechanical models for IHI-ST. For a description of these parameters, see appendix A

Reference	Peak angular velocity ω (rad/s)	Peak angular acceleration α (rad/s^2^)	Peak linear acceleration (m/s^2^)	Remarks
Duhaime et al. [2]	61	1138	91	Range is for different neck types
			56-136	
Jenny et al. [3]	153	13252	271 (center of gravity)	
			665 (top of head)	
Cory & Jones [4]	61 (mean: 51)	10216 (mean: 8693)	1736 (mean: 1488)	Largest values for gravity assisted shaking. Mean is averaged over different parameter combinations tried.
Prange et al. [5]	28	2640 (mean: ~4000)		Values for typical example given. Mean is value averaged over subjects
Cheng et al. [6]	N/A			
Lloyd et al. [7]	35	1587	74	Values for two different dolls
	25	1068	97	
Cirovic et al. [8]	25	650	45	Values for P3/4 test dummy and proprietary doll
	40	1180	76	
Koizumi et al. [9]	N/A			
Yamazaki et al. [10]			46-60	Range is for different shaking styles
Miyazaki [11]	N/A			
Tomlinson & Taylor [12]	N/A			
Jenny et al. [13]	80-106	9613-13260		Ranges are peak values for different trials

##### Mathematical models

RBM studies are similar to physical modeling studies in the sense that an IHI-ST event is applied to the model, often shaking applied to the torso, and the resulting head dynamics are calculated using the RBM (Table 4). Both Wolfson et al. [14] and Lintern et al. [17] reported that the largest accelerations in their models occur during chin-to-chest or occiput-to-back endpoint contacts.

**Table 4:** Values of kinematic parameters as reported in papers on mathematical RBMs for IHI-ST. For a description of these parameters, see appendix A

Reference	Peak angular velocity ω (rad/s)	Peak angular acceleration α (rad/s2)	Peak linear acceleration (m/s2)	Remark
Wolfson et al. [14]	20	<< 1000		Values when no head-torso impact takes place
	~195	10000		Values when head-torso impact takes place
Bondy et al. [15]	45	18567		Values for two different neck stiffness
	39	21205		
Jones et al. [16]	17	1133	96 80-350	Range is for different stiffness properties of neck
Lintern et al. [17]	~20		200-250	Lamb model

#### Step 4. Skull-internal transfer

##### Physical models

Only a few studies used dolls with detailed internal head anatomy [6, 9–12]. These dolls contained, for example, bone structures obtained from CT-scans or models for cerebro-spinal fluid (CSF), brain, or eyes. In these studies, water was used to model CSF, gelatin or silicone gel to simulate brain tissue, and agar gel to represent internal eye structures.

##### Mathematical models

RBMs described in the literature focus on gross body dynamics and do not address internal transfer within the skull. FEMs, however, explicitly focus on modeling the mechanical behavior of micro-structures, such as stresses and strains in the brain and bridging veins or local pressures in the retina. For this purpose, attention has been devoted to modeling techniques for CSF [6, 24–26, 28]. In some studies, the influence of anatomical features such as the fontanelle [6, 21, 26, 29] and the size of the subarachnoid space [22] on the amount of movement and deformation of the brain was qualitatively investigated. For all FEM studies, having representative material properties and detailed geometrical data for all anatomical structures in the head is quintessential. For this purpose, material properties from the literature are generally used in such studies. Batterbee et al. [29] applied another approach and did a sensitivity analysis to identify parameters to which FEM output was most sensitive.

#### Step 5. Internal dynamics

Both physical and mathematical models on IHI-ST studying motion, deformation, and forces acting on the structures inside the head are generally focused at a limited number of phenomena, traditionally associated with IHI-ST:

- stretching of bridging veins related to subdural hematoma (SDH),
- internal pressure distributions within the brain related to diffuse axonal injury (DAI),
- peak stresses within the eye related to retinal hemorrhaging (RH).

##### Physical models

Several studies investigated stretching of bridging veins during IHI-ST by placing markers onto a surrogate brain and on the skull [6, 9, 11]. By tracking the relative positions of these markers during IHI-ST experiments, the stretching of bridging veins was estimated. In Cheng et al. [6], marker excursions in the order of 2.5-3mm were reported. Peak values for bridging vein stretch ratios (stretched length divided by original length, see Appendix A) found by Miyazaki [11] ranged from 4 to 5 and the ones found by Koizumi et al. [9] ranged from 1.5 to 3.

Stresses within the eye during IHI-ST were measured using a pressure sensor inside an eye model in Yamazaki et al. [10], showing peak compressive stresses up to 0.85kPa and peak tensile stresses up to 0.62kPa, both at the posterior pole of the eye and averaged across their subjects shaking the doll. Using a 2D representation of a sagittal slice of the head, internal stress distributions during machine based shaking was visualized using a photo-elastic material in Tomlinson & Taylor [12]. They reported peak shear stresses at the brainstem of 1.15kPa.

##### Mathematical models

In Batterbee et al. [26] it was qualitatively stated that the presence of a fontanelle could increase the likelihood of rupture of bridging veins. Couper & Albermani [28] showed data with peak stretch ratios of bridging veins up to approximately 2.15 and Morison [18] found bridging veins stretch ratios up to 1.25 during a simulated IHI-ST event. Raul et al. [22] investigated the effect of the size of the subarachnoid space on bridging vein stretch ratios, reporting peak ratios in the vertex area of 1.9 for normal and 1.22 for larger sized subarachnoid spaces. In sensitivity analyses performed by Batterbee et al. [29], bridging vein stretch ratios were found to range between 1.2 for pure translational motion up to 3 for combined translational and rotational movement. Values depended on choice of model parameters such as material properties and CSF layer thickness. Roth et al. [20] reported maximum bridging vein stretch ratios of 1.9 in their simulations and a maximum pressure of 22kPa at the frontal area of the brain. Using a two-step approach, Coats et al. [31] first validated their FEM for piglet brain by comparing model outputs to experimental data on piglet brain deformation and brain-skull displacement. They reported that for a good fit the model required adjusting the brain stiffness, which was derived from *in vivo* experiments, by a factor 1.5-2.25, suggesting that porcine brain is stiffer *in situ* than *in vivo*. Next, threshold values for injury were determined for their FEM by comparing model outputs to experimental data on porcine intracranial hemorrhaging during IHI-ST experiments.

In Cheng et al. [21] it was reported that the highest stresses (Von Mises stresses about 1MPa) in the brain appear near the fontanelle in their FEM. Couper & Albermani [28] provide detailed analyses of brain strains in their 3D FEM during IHI-ST. High strains were developed in contact points between frontal lobe and cranium, occipital lobe and sinus confluence, and in the inferior sagittal sinus. Besides this, high strains manifested in corpus callosum and in connections between brainstem and cerebrum. Peak values for strains reported in [28] were 0.7 (Tresca strains). Morison [18] observed brain stresses to increase from brainstem to vertex, with maximum values of 0.8 kPa. Ponce & Ponce [30] found the largest displacements in the central areas of the brain and a more or less uniform distribution of stresses across the brain.

In exploring the response of their eye FEM, Cirovic et al. [19] reported displacements accumulating up to 0.8 mm in the center of the eye and a maximum stress of 12MPa at the orbit when the model was driven by its characteristic frequency of 200Hz. Hans et al. [23] reported mean retinal nodal forces (forces at key points of the FEM) of 0.05-0.08N with peaks up to 0.45N. In the eye FEM from Nadarasa et al. [33] pressures of 1.5-2kPa at the posterior pole of the eye, extending to the mid retina were found. Rangarajan et al. [27] reported stresses in the eye of 14-120kPa, depending on modeling techniques, with the largest stresses found at the interface between the retina and vitreous body. Yoshida et al. [32] reported that the location of maximum stress in their IHI-ST simulations was at the posterior pole of the eye. In their data, the maximum normal component of stress was approximately 1kPa.

#### Step 6. Injury thresholds

In order to assess whether values for kinematic and dynamic quantities obtained in modeling studies can lead to actual injury, some estimates of injury thresholds for IHI-ST must be made. Because threshold data on infant biological material is very scarce, most physical and mathematical modeling studies rely on threshold values derived from literature values originally not obtained from infants. Only Morison [18] presented own original research specifically aimed at obtaining data on bridging veins threshold values for infants.

##### Physical models

In many IHI-ST studies, injury thresholds are mainly referred to in order to estimate whether the measured head accelerations could be hazardous. For this purpose, many studies use plots of (peak) angular acceleration vs (peak) angular velocities (henceforth called α-ω plots), an injury criterion originally used in head impact studies. In order to be applied to IHI-ST events, injury thresholds from different sources in animal impact literature have been scaled down for use on infants (Table 5). The use of α-ω plots or scaled data from animal experiments has been criticized in several studies and it has been suggested that injury mechanisms, and hence injury thresholds, might be different for IHI-ST as compared to events with impact [4, 10, 13–15, 18, 25, 28]. Alternatively, Cory & Jones [4] and Lloyd et al. [7] used the head-injury-criterion (HIC) using threshold values by Van Ee et al. [36] and Stürtz [37]. HIC is a measure to assess the likelihood of injury to occur as a consequence of impact originating from studies in automotive safety research. It is computed by integrating measured accelerations over short time periods (typically a few tens of milliseconds).

**Table 5:** Threshold values for injury criteria used in physical models for IHI-ST

Reference	Threshold type	Threshold value	Threshold source
Duhaime et al. [2]	α-ω plots	Concussion: α > 10.000, ω > 100 SDH: α > 37.000, ω > 120 DAI: α > 40.000, ω > 250	[48]
Jenny et al. [3]	N/A		
Cory & Jones [4]	α-ω plots	Concussion: α > 6.000, ω > 58 ([49]) α > 3.000, ω > 45 ([50], 50% chance of concussion) SDH: α > 22.500, ω > 70 ([49]). Also values from [2].	[2, 49, 50]
	HIC	HIC > 840 for children	[37]
Prange et al. [5]	α-ω plots	N/A	[51–54]
Cheng et al. [6]	N/A		
Lloyd et al. [7]	HIC-15, α for bridging vein rupture	HIC-15 > 390 α > 10.000	[36] [39]
Cirovic et al. [8]	N/A		
Koizumi et al. [9]	Bridging vein stretch ratio	Stretch ratio > 1.5	[38]
Yamazaki et al. [10]	N/A		
Miyazaki [11]	Bridging vein stretch ratio	Stretch ratio > 3	[39]
Tomlinson & Taylor [12]	Shear stress	Shear stress > 20 kPa	N/A
Jenny et al. [13]	α-ω plots	N/A	[50, 52]

In the few physical model studies addressing internal head dynamics, threshold values for bridging vein rupture were taken from *in situ* experiments on cadaveric human bridging veins by Lee & Haut [38] and a cadaver impact study by Depreitere et al. [39] (Table 5).

##### Mathematical models

RBM studies focusing on gross head dynamics rely on similar sources for injury thresholds as the physical modeling studies described before (Table 6). For FEM studies, however, much more detailed injury thresholds are required: detailed limits for the stresses and strains that the biomaterials can withstand.

**Table 6:** Threshold values for injury criteria used in mathematical models for IHI-ST

Reference	Threshold type	Threshold value	Threshold source
Rigid body models			
Wolfson et al. [14]	α-ω plots	Same as [4]	[2, 4, 49, 50]
Bondy et al. [15]	α-ω plots		[2, 4, 50]
Jones et al. [16]	α-ω plots	N/A (Fig 6 is wrong)	[2, 49, 50]
Lintern et al. [17]	N/A		
Finite element models			
Morison [18]	Bridging vein stretch ratio	Ratio 1.5 may be too large for children. Might be 1.15	[38, 55, 56] Own research
Cirovic et al. [19]	N/A		
Roth et al. [20]	Bridging vein stretch ratio	N/A	[38]
Cheng et al. [21]	N/A		
Raul et al. [22]	Bridging vein stretch ratio	N/A	
Hans et al. [23]	Retinal adhesive force	0.14N	[42, 43]
Couper & Albermani [24]	N/A		
Couper & Albermani [25]	N/A		
Batterbee et al. [26]	N/A		
Rangarajan et al. [27]	N/A		
Cheng et al. [6]	N/A		
Couper & Albermani [28]	Bridging vein stretch ratio Strain leading to Axional Injury	1.5 0.1	[38] [40]
Batterbee et al. [29]	N/A		
Ponce & Ponce [30]	Von Mises stress	0.048 N/mm^2^: 50% injury chance 0.080 N/mm^2^: 100% injury chance	[41]
Coats et al. [31]	Pia-arachnoid complex stretch ratio	1.31	Own research
	Principal stress at brain surface	45.4 kPa	
Yoshida et al. [32]	N/A		
Nadarasa et al. [33]	N/A		
Other models			
Bandak [34]	Neck distraction force	209 N (baboon) 249 N (goat) 445 N (human neonate)	[44] [45, 46] [47]

FEM studies investigating the possibility of SDH in IHI-ST used data from Lee & Haut [38] and Depreitere et al. [39] for bridging vein stretch ratio limits. Morison [18] was one of the very few specifically measuring these stretch ratio limits for infants in a small sample of pediatric bridging veins and suggests that tolerance levels for these veins might be lower than values reported for larger and higher age ranges in literature. Threshold values for tissue strains leading to DAI in Couper & Albermani [28] were derived from *in vitro* animal experiments by Morrison et al. [40], while Ponce & Ponce [30] used brain shear stress thresholds that were not measured, but estimated by Meyer et al. [41] through numerical reconstruction of real-life cases. In their FEM used to study the likelihood of RH during IHI-ST events, Hans et al. [23] used threshold values for the retinal adhesion force from monkey eyes experiments by Kita & Marmor [42, 43]. Alternatively, Coats et al. [31] determined threshold values for intracranial hemorrhaging and principal stress at the surface of the brain in a piglet brain FEM using an optimizing approach. Reported injury threshold values of 1.31 for pia-arachnoid complex stretch ratio and 45.4kPa for principal stress at brain surface yielded the best match between FEM predictions and experimental data on piglet intracranial hemorrhaging due to IHI-ST events.

Bandak [34] estimated the limit strength of an infant’s neck based on several animal studies [44–46] and an old study in which the force required to dissever the head was measured in a number of fetuses [47].

Besides forces and deformations exceeding injury thresholds, several studies suggest that other effects might play a role in causing injury in IHI-ST. Most notably, it has been suggested that repetitive IHI-ST events can lead to an accumulation of displacements and stresses in the eye [19, 23, 27] and in the head [6, 9, 14]. Besides this, some studies have suggested that, due to the anatomy of the head, rotational motion might be more damaging than translational motion of comparable intensity [18, 29, 33]. This is supposedly because the CSF protects the brain from sudden accelerations against the skull by providing a cushioning effect in translational motion, while the same CSF provides lubrication and hence promotes free brain movement in rotational motion. Finally, both Couper& Albermani [28] and Miyazaki [11] have pointed to the reverse rotational motion between brain and skull following chin-to-chest or occiput-to-back endpoint contacts. In Miyazaki [11] this is indicated as the most important mechanism for brain injuries.

#### Step 7. Injury

In IHI-ST literature, different types of statements are made to draw conclusions concerning the likelihood of injury occurring as a consequence of impactless events, such as shaking. Broadly speaking, these statements can be divided into the following three categories.

- Threshold comparison: data obtained from simulated IHI-ST events is compared to injury threshold values to make a statement on the likelihood of injury in IHI-ST.
- Comparison with other activities: by comparing data from simulated IHI-ST events to data obtained in simulated falls, simulated impact events or daily activities an estimate is made of the relative likelihood of injury in IHI-ST as compared to another activity.
- Qualitative conclusion: A qualitative opinion, without reference to injury thresholds, is expressed about the likelihood of occurrence of injury in IHI-ST events in connection with the research presented in the paper.

Table 7 shows that using mechanical models for IHI-ST and threshold comparison with injury criteria based on α-ω plots and bridging vein stretch ratios from literature, a number of researchers have expressed strong opinions concerning the possibility of IHI-ST causing fatal injury or subdural hematoma. For mathematical models (Table 8), especially FEMs, often more cautious qualitative conclusions are drawn, pointing out mechanisms that might be important for understanding IHI-ST.

**Table 7:** Injury related conclusions in papers on physical models for IHI-ST. Abbreviations used in the table: SDH: subdural hematoma, BV: bridging veins, RH: retinal hemorrhage, RBM: rigid body model, DAI: diffuse axional injury, CSF: cerebro-spinal fluid. See main text for definitions of the ”Types of statements”

Reference	Types of statements	Study conclusions with respect to injury
Duhaime et al. [2]	Threshold comparison Comparison with impacts	IHI-ST, at least in its most severe form, is not usually caused by shaking alone
Jenny et al. [3]	Qualitative	Angular accelerations found are larger than those of Duhaime et al. [2].
Cory & Jones [4]	Threshold comparison	It cannot be categorically stated that ‘pure shaking’ cannot cause fatal head injuries in an infant.
Prange et al. [5]	Threshold comparison	There is no data to support that α-ω values during shaking and impact against a padded surface are sufficient to cause trauma in an infant .
	Comparison with impacts	α-ω values are larger in impacts than in shaking and falls, therefore, inflicted impacts may be more frequently associated with inertial brain injury.
Cheng et al. [6]	Qualitative	A skull with open fontanelle may be more vulnerable to shaking than a closed skull.
Lloyd et al. [7]	Threshold comparison	Aggressive or resuscitative shaking is not likely to be a primary cause of DAI, primary RH or SDH in a previously healthy infant.
	Comparison with activities during daily living	Head kinematics during aggressive shaking of a doll was indistinguishable from those of a 7 month infant during activities during daily living.
Cirovic et al. [8]	Qualitative	Blood pressure build-up in the head during shaking might contribute to eye hemorrhaging observed in IHI-ST.
Koizumi et al. [9]	Threshold comparison	SDH is likely to occur as a result of shaking at a frequency of 3 Hz and amplitude of 50 mm. Lower frequencies do not lead to SDH due to BV breaking.
Yamazaki et al. [10]	Comparison with falls	The time integral of eyeball stresses during a cycle of shaking is larger than during a fall. This might explain why RH is more frequent in IHI-ST than in falls.
Miyazaki [11]	Threshold comparison	Relative displacement of brain with respect to skull exceeds BV rupture thresholds during shaking in most cases.
	Comparison with falls	Relative displacements are larger for shaking than for low height falls.
Tomlinson & Taylor [12]	Threshold comparison	Maximum shear stresses measured during shaking are much smaller than values typically required for permanent brain damage to occur.
Jenny et al. [13]	Qualitative	Higher angular accelerations and velocities, due to chin-chest contact, measured in this study, suggest a higher potential for injury in shaking than previously reported

**Table 8:** Injury-related conclusions in papers on mathematical models for IHI-ST. The abbreviations are the same as those used in Table 7. See main text for definitions of the ”Types of statements”

Reference	Types of statements	Study conclusions with respect to injury
Rigid body models		
Wolfson et al. [14]	Threshold comparison	Head impact is required in RBM to exceed injury criteria.
	Qualitative	Research should be focused on specific injury mechanisms in low-energy cyclic loading.
Bondy et al. [15]	Qualitative	Results of RBM are consistent with other biomechanics studies on IHI-ST.
Jones et al. [16]	Qualitative	Head acceleration in the presented model compare to those from doll experiments. Neck stiffness properties are important determinants of peak vertex accelerations.
Lintern et al. [17]	Qualitative	RBM can reproduce head kinematics during in vivo lamb shaking and can describe complex head-torso impact, which give dominant accelerations in IHI-ST.
Finite element models		
Morison [18]	Threshold comparison	IHI-ST could produce bridging vein strains close to thresholds for failure and should be considered as a possible cause for SDH. Bridging vein threshold stretch ratio of 1.5 might be too large for children.
	Qualitative	Rotational component of movement accounts for 93% of bridging vein strains.
Cirovic et al. [19]	Qualitative	Interaction between the eye and intra-orbital fat determines the eye motion in high acceleration situations. Resonance effects may lead to build-up of stresses and displacements during shaking.
Roth et al. [20]	Comparison with impacts	Vigorous shaking can have the same consequences as an impact in terms of SDH.
Cheng et al. [21]	Qualitative	The presence of open fontanelle could increase the chance of SDH due to shaking.
Raul et al. [22]	Qualitative	Enlargement of the subarachnoid space has a damping effect due to greater amount of CSF and reduces relative brain-skull displacement. Benign enlargement of subarachnoid space may not be a risk-factor for SDH.
Hans et al. [23]	Threshold comparison	Shaking alone maybe enough to cause RH since there are more sustained and higher forces than in fall-caused impacts.
	Comparison with impacts	The optic nerve causes more localized stresses in shaking than in impact.
Couper & Albermani [24]	Qualitative	Modeling CSF as a fluid is important for modeling IHI-ST. The volume of CSF and CSF layer thickness variations strongly affect brain-CFS interaction.
Couper & Albermani [25]	Qualitative	The brain-CSF interaction in modeling IHI-ST depends on the volume of CSF and thickness variations of the gyri. Gyri protusions alleviate deep brain stress concentration and hence aid injury mitigation.
Batterbee et al. [26]	Qualitative	Fontanelle reduces effectiveness of buoyancy forces, which normally cushion the brain and therefore increases the chance on SDH. Larger internal brain stresses due to fontanelle could also increase the likelihood of other brain damage.
Rangarajan et al. [27]	Qualitative	Areas of maximum stress in the eye model correlate with clinical manifestations of RH at the ora serrata and posterior pole. Stresses build up over multiple shaking cycles.
Cheng et al. [6]	Qualitative	Special features of infant skulls, such as fontanelle, are fundamentally important to understand how the head behaves when shaken.
Couper & Albermani [28]	Threshold comparison	Shaking an infant leads to a specific pattern of brain motion, increased likelihood of focal axonal injury at deep brain regions and at locations of brain-skull contact, and a capacity for development of SDH due to bridging vein rupture.
Batterbee et al. [29]	Qualitative	Sensitivity of the model outputs to parameters values depends on the shaking conditions. Particularly, density ratio, CSF thickness and fontanelle size have sensitivity that depends on excitation type because they affect buoyance effects, which are more dominant in translational than in rotational excitation.
Ponce & Ponce [30]	Qualitative	FEM appears to be a practical, universal, economical and fast tool with important forensic use.
Coats et al. [31]	Qualitative	Intercranial hemorrhage in piglets is best predicted by a model containing spring connectors to represent pia-arachnoid complex. Top 1% peak connector strains are best predictor of intercranial hemorrhage.
Yoshida et al. [32]	Comparison with impacts	The time integral of stress in the eye model could be a good predictor of RH during IHI-ST. It is larger in a single cycle of shaking than in a single impact event.
Nadarasa et al. [33]	Comparison with falls	Pressure and stress in the eye are 4x and 14x higher in shakes than in falls. RH in infants is more likely due to rotational than due to linear accelerations. Shaking is more dangerous than domestic falls.
Other		
Bandak [34]	Threshold comparison Comparison with falls	Head dynamics in IHI-ST generate forces that are far too great for infant necks to withstand without injury. Shaking head velocity corresponds to a free fall of 1 m.

## Discussion

In this paper we set out to obtain an overview of systematically review mathematical and physical models developed for understanding IHI-ST. Pioneering research initially focused on measuring head kinematics during IHI-ST events in mechanical surrogates and RBMs, using injury criteria based on α-ω plots (steps 1-3 in Fig. 2). Later, research shifted towards creating more detailed mathematical FEMs and physical models containing representations of infant brain or eye structures (steps 4-7 in Fig. 2). For those models, injury criteria based on material properties were used. Without wanting to sell these studies short, which indeed have made various significant contributions to our understanding of possible mechanisms underlying IHI-ST, it must be said that virtually all models rely on (scaled) non-infant data for both material properties and injury threshold values. It remains essentially unknown to what extent properties of infant biological materials are comparable to adults or experimental animals. Therefore, in our opinion, there is an urgent need to better validate material properties and injury threshold in current state-of-the-art mathematical and mechanical models for IHI-ST against infant macroscopic (e.g. infant neck stiffness) and microscopic data (e.g. failure stretches of infant bridging veins). We understand that this is a difficult task due to ethical reasons and scarcity of research material, but nevertheless the yield of such research might be substantial.

Besides the fact that injury thresholds are derived from non-infant data, currently used injury thresholds may also be unsuitable for IHI-ST because of the injury mechanisms originally applied to determine the thresholds. Several recent modeling studies have suggested that injury mechanisms in IHI-ST might differ from that during impact, pointing out e.g. build-up of blood and CSF pressure over multiple shaking cycles and resonance effects. Furthermore, a shaking cycle in IHI-ST is a relatively low intensity, long duration exertion of force when compared to an impact, which is a high intensity, short duration force peak. These differences between shaking and impact may call for a re-evaluation of impact-based injury thresholds used in studies focusing on head kinematics in IHI-ST. In this respect, comparison of head kinematics during IHI-ST with kinematics during daily activities, as in Lloyd et al. [7] could yield valuable new insights. Besides this, it might be worthwhile to compare injury criteria based on the transfer of energy or momentum during IHI-ST with those during impact.

## Key points

1. In virtually all mechanical and mathematical IHI-ST models, injury thresholds are derived from scaled non-infant data. Studies focusing on head kinematics often use injury thresholds derived from impact studies.
2. Physical modeling studies, as well as some RBMs focusing on head kinematics, often conclude that IHI-ST cannot produce injury.
3. Physical models containing detailed head anatomy, as well as FEM studies of infant head and eye, however, suggest, sometimes qualitatively, that IHI-ST events can indeed produce injuries such as bridging vein rupture and retinal hemorrhaging.
4. Research aimed at investigating failure thresholds of infant biological material is called for, as well as research into possible alternative injury mechanism and alternative injury criteria for IHI-ST.

## Electronic supplementary material

ESM 1 (PDF 1075 kb)
