# Supplementary material for: Modeling of inflicted head injury by shaking trauma in children: what can we learn? Part II: A systematic review of mathematical and physical models
Source: Forensic Sci Med Pathol. 2019 Feb 20;15(3):423–36. doi: 10.1007/s12024-019-00093-7 (PMC6687692; doi:10.1007/s12024-019-00093-7)
Supplement: Supplementary file 1 — (PDF 1075 kb) [file 12024_2019_93_MOESM1_ESM.pdf]

# Appendix A: basic concepts in (bio)mechanics

**Kinematics** describes the movement of an object, such as e.g. an infant's head, by means of specifying its position (location and orientation), velocity and acceleration over time. In case of the object **translating** (changing location) from left to right in Figure A1, its translational (or linear) velocity $v_l$ is the rate of change of the position $x$ over time $t$:

$$v_l = \frac{dx}{dt}$$

and acceleration $a_l$ is the rate of change of the velocity $v$ over time:

$$a_l = \frac{dv_l}{dt}.$$

Usually, $x$ is measured in meters (m), $v_l$ is measured in meters per second (m/s) and $a_l$ is measured in m/s$^2$. In Figure 1, the lengths of the arrows indicate the magnitude of a speed or acceleration. In case of a constant linear acceleration $a_l$ pointing right, not only the position of any point on the object changes, but the speed of any point on the object will also increase between Time 1 and Time 2, making the distance traveled in the next step even bigger. If the object does not deform and there is only linear (translational) motion, all points on the object experience the same speed and acceleration.

In case of **rotational** movement (changing orientation), position, velocity and acceleration can also be expressed in angular coordinates. In the rotation example in Figure 1, the position of the head is specified by its angle $\theta$ in radians. In angular coordinates, velocity is expressed as the rate of change of the angle: angular velocity ($\omega$ in rad/s) and similarly the angular acceleration $\alpha$ (in rad/s$^2$) is the rate of change of the angular velocity.

If the object rotates around a point (point O in Figure 1) and there is no translation, all points on the object re-orient at the same rate because they experience the same angular velocity and angular acceleration. However, all points except the center of rotation O experience translation, with points located at a larger distance $r$ from point O translate more and faster than points closer to point O. This is because points located further away from O travel along larger circles and thus have to travel a larger distance with each rotation.

Consider an arbitrary point on the circle in the bottom left section of Figure 1, located at a distance $r$ from point O at angle $\theta_1$ at Time 1. At that instance there is an angular velocity $\omega_1$ and a constant angular acceleration $\alpha$. Due to the angular velocity, the point on the circle ends up at angle $\theta_2$ by traveling along the circle perimeter, while the angular velocity increases to $\omega_2$ due to the angular acceleration.

All points of the object, except point O (which only rotates), translate along a circular path around point O. The radius of that circular path is defined by the distance $r$ of the point from O. The translational velocity with which the point travels along that circular path is called the tangential velocity and is related to $\omega$ and $r$ by:

$$v_t = \omega \cdot r.$$

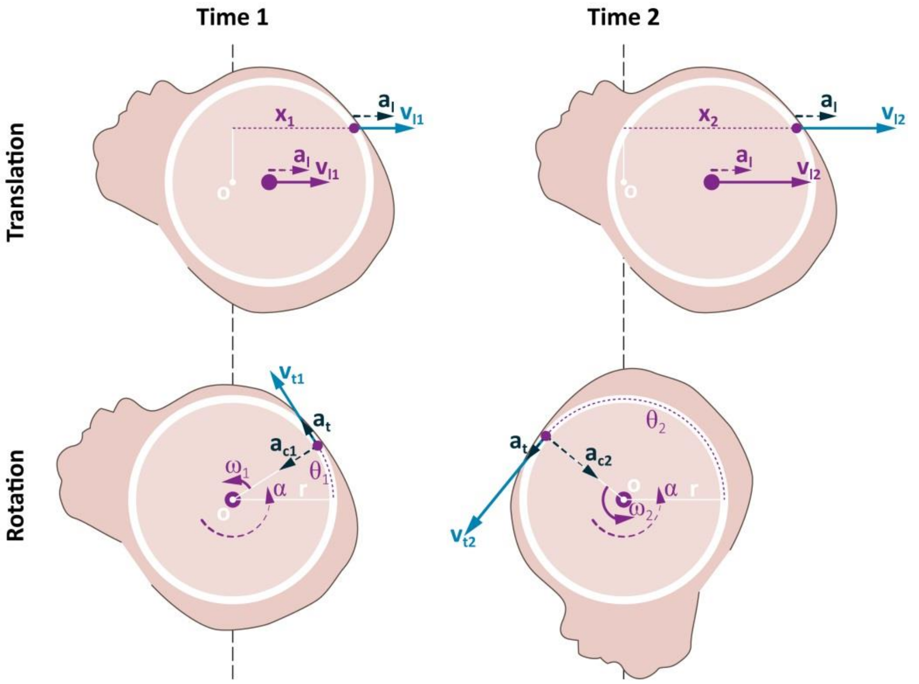


**Figure A1: Kinematics of an infant head, showing (top row) the translational (or linear) velocities and accelerations occurring during pure translation of the head, and (bottom row) the angular velocities and accelerations and translational (or linear) velocities and accelerations occurring during pure rotation of the head. The lengths of the arrows indicate the magnitude of the velocities and accelerations. Symbols are explained in the main text. Subscripts 1 and 2 are used to indicate values of variables at Time 1 (left) and Time 2 (right), respectively, which are two arbitrary, successive moments in time.**

Consequently, as the angular acceleration $\alpha$ increases the angular velocity from $\omega_1$ at Time 1 to $\omega_2$ at Time 2 and $r$ is constant, the point on the perimeter of the circle also experiences a translational acceleration $a_t$ defined by:

$$a_t = \frac{dv_t}{dt} = \frac{d\omega}{dt} \cdot r = \alpha \cdot r,$$

which increases the tangential velocity from $v_{t1}$ at Time 1 to $v_{t2}$ at Time 2. As shown in Figure 1, the tangential velocity not only changes in magnitude, but also in direction, as it travels along the circular path. The point moving along the circle would, if not connected to the object, move away from point O in the direction of $v_t$. However, because the point on the circle is connected to the object, it is constantly being pulled towards point O, as if it was a ball tied to a rope that is being swung around. The magnitude of this translational acceleration in radial direction, better known as the centripetal acceleration $a_c$ is defined by:

$$a_c = \frac{(v_t)^2}{r} = \omega^2 \cdot r.$$

Summarizing, for an object rotating as a non-deforming whole around a center of rotation:

- points on the object further away from the center of rotation experience larger translational velocities and translational accelerations than points closer to the center of rotation,
- the rotational velocity and angular acceleration are identical for all points along the object.

**Dynamics** deals with the description of forces acting on an object and the behavior of deformable objects under loading. In this brief introduction, we will limit ourselves to elastic structures. In biomechanics, including IIHII modelling, such spring-like structures play an important role.

Elastic behavior is characterized by the amount of pressure applied to a structure (called stress) and the consequential length-change or deformation due to this pressure. In the literature, the most common ways to express this length change are by means of *stretch ratio* and *strain*. The stretch ratio $\lambda$ is simply the new length divided by the original length: $\lambda = \frac{l}{l_0}$, while strain $\varepsilon$ is the length change as a percentage of the original length: $\varepsilon = \frac{\Delta l}{l_0} = \frac{l - l_0}{l_0}$. For example, if a bridging vein initially has a length of 2 cm and is stretched to a length of 2.5 cm it's stretch ratio = 2.5 cm/2 cm = 1.25 and it's strain = 0.5 cm/2cm = 0.25 = 25%. In the main part of the paper, we will exclusively use stretch ratios.

## Appendix 2 – Search Queries used to find papers describing aninal, mechanical and mathematical models for IIHII

**Q1.** ((finite[All Fields] AND ("elements"[MeSH Terms] OR "elements"[All Fields] OR "element"[All Fields]) AND shaken[All Fields]) OR (((biomechanical[All Fields] AND shaken[All Fields]) OR (("models, animal"[MeSH Terms] OR ("models"[All Fields] AND "animal"[All Fields]) OR "animal models"[All Fields] OR ("animal"[All Fields] AND "model"[All Fields]) OR "animal model"[All Fields]) **AND** ("shaken baby syndrome"[MeSH Terms] OR ("shaken"[All Fields] AND "baby"[All Fields] AND "syndrome"[All Fields]) OR "shaken baby syndrome"[All Fields] OR ("shaken"[All Fields] AND "baby"[All Fields]) OR "shaken baby"[All Fields]))) OR (non[All Fields] AND accidental[All Fields] **AND** ("craniocerebral trauma"[MeSH Terms] OR ("craniocerebral"[All Fields] AND "trauma"[All Fields]) OR "craniocerebral trauma"[All Fields] OR ("head"[All Fields] AND "injury"[All Fields]) OR "head injury"[All Fields]) AND model[All Fields]))) OR (("Simulation"[Journal] OR "simulation"[All Fields]) AND shaken[All Fields])

**Q2.** (((((((biomechanical) OR animal model) OR finite element) OR simulation) OR mannequin) OR dummy)) **AND** (((shaken baby) OR abusive head trauma) OR non accidental head)

**Q3.** (((Biomechanical Phenomena/methods [Mesh]) OR ((((((biomechanical model) OR biomechanical evaluation) OR biomechanical study) OR biomechanical) OR biomechanical analysis) OR "Models, Neurological"[Mesh]) OR "Models, Theoretical"[Mesh]))) **AND** (((((((((((Hematomas, Subdural) OR Subdural Hematomas) OR Subdural Hematoma) OR Hemorrhage, Subdural) OR Hemorrhages, Subdural) OR Subdural Hemorrhage) OR Subdural Hematoma, Traumatic) OR Subdural Hemorrhages) OR Hematoma, Traumatic Subdural) OR Hematomas, Traumatic Subdural) OR Traumatic Subdural Hematoma) OR Traumatic Subdural Hematomas)

**Q4.** (((biomechanic* OR dynamic* OR kinematic* OR motion OR force OR impact) AND (phenomena OR method OR model OR evaluation OR study OR analysis)) OR ("finite element" OR "FEM") OR ((animal OR neurological OR theoretical) AND model) OR simulat* OR doll OR mannequin OR dummy OR anthropomorphic) **AND** ((shake* AND (infant OR baby OR impact)) **AND** ("subdural Hematoma" OR "subdural Hemorrhage" OR ((craniocerebral OR head OR retinal) AND (injury OR trauma OR bleeding))) **AND** (("non accidental" OR "nonaccidental" OR "non-accidental") OR inflict* OR violen* OR abus* OR shaking))

Pubmed was searched using queries Q1 to Q4 and combining their results. Scopus was searched using query Q4.

Appendix III - Data Extraction Tables

| General | | | | Model description | | | | | Model input | | | | | | Kinematic response | | | | Dynamic response | | | | | Injury criterium | | | Conclusions | Remarks |
|---|---|---|---|---|---|---|---|---|---|---|---|---|---|---|---|---|---|---|---|---|---|---|---|---|---|---|---|---|
| Authors | Title | Reference | Aim | Type of model | Modelled entities | Mat. props | Repr. Age | Repr. Weight | Type of shaking | Number of subjects shaking | Instructions to subjects | Duration of shaking | Frequency of shaking | Amplitude of shaking | (peak) linear acceleration | (peak) linear velocity | (peak) angular acceleration | (peak) angular velocity | skull | eye | brain | veins | neck | type of cryterium | source of tresholds | references | | |
| Cheng, J.,Howard, I. C.,Rennison, M. | Study of an infant brain subjected to periodic motion via a custom experimental apparatus design and finite element modelling | J Biomech 43 (2010) 2887-96 | Estimate brain motion and bridging vein streches | Physical | Spherical skull (diameter=10 cm), fontanel (size=4cm), Single volume brain, cerebrospinal fluid (5mm), bridging veins (marker positions), includes blood circulation | Given in Table 2 | N/A. Model is compared to initial experiment s with 9 mo TRL crash dummy | N/A. TRL crash dummy body mass is 9.5 kg | Machine shaking | | | | ~ 3 Hz (Fig 9) | | | | | | | | | Markers move 1-2 mm during shaking | | | | | Qualitative statement that skull with fontanel might be more vunerable to IHI-STthan closed skull. | Suggestion that resonance effect might play role in IHI-ST. |
| Cirovic, Srdjan Freddolini, Marco Goodwin, Rachel Zimarev, Daniel | Shaken Mannequin Experiments: Head Motion Pattern and Its Potential Effect on Blood Pressure | Journal of Forensic Biomechanics 3 (2012) 1-4 | Estimate head motion and blood pressure fluctuations in head during shaking | Physical. P3/4 and prop model. | Prop: Head (diameter = 9,2 cm, weight = 480 g ), neck flexible link (4 cm for prop), torso. | For prop: torso=wood, head=hard plastic, neck=silicon rubber. No material props given. | P3/4: 9 mo. | P3/4: body 9 kg. Prop: body 1.5 kg | Human shaking | 10 (5 male, 5 female) (head kin expt). 9 (6 m ale, 3 female) (blood pressure expt) | "as violently as possible" | 10 s for prop, 5 s for P3/4 | 3.9 averaged for both dolls(page 3) | | X-direction: 45 m/s^2 (P3/4), 76 m/s^2 (prop). Z-direction: 30 m/s^2 (P3/4), -50 m/s^2 (prop). (Fig 3). Note: there is a net linear acceleation in negative Z-direction for prop. 45 m/s^2 (P3/4), 76 m/s^2 (prop) (Table 1) | | 650 rad/sec^2 (P3/4), 1180 rad/sec^2 (prop) (Table 1) | 25 rad/s (P3/4) 40 rad/s (prop) (Table 1) | | | | blood presure is increased in prop during shaking. dP 25-35 +/- 10 mmHG, dPmax 65-60 +/- 15 mmHg | | | | | increased bloodpressure and negative g effect in prop during shaking might contribute to eye haemorraging often found in IHI-ST | |
| Cory, C. Z. Jones, M. D. | Can shaking alone cause fatal brain injury? A biomechanical assessment of the Duhaime shaken baby syndrome model | Medicine, Science and the Law 43 (2003) 317-333 | Parametric study of effects of neck type and head weight on angular accelerations during shaking. | Physical. | Head (weight=830 gr; diameter ~10 cm), neck single hinge and neck flexible link (length = 4 cm), torso. | Flexible neck: red hollow tubing (Harris-Scientific, Cardiff). Torso: cotton and metal. Paper states that model parameters chosen in accordance to those of Duhaime et | N/A. Initial experiment s on models with mean masses of 1 mo, 7 mo and 18 mo | Head 830 g. Body mass 3-4 kg | Human shaking | 1(initial experiments: 11 volunteers, 7 female, 4 male) | Shaking in anterior-posterior direction. Gravity assisted shaking. (Saggital plane) | | | | 1736 m/s^2 (max value) 1488 m/s^2 (averaged over parameter combinations) (table 3) | 10.3 m/s (max value), 8.6 m/s (averaged over parameter combinations) (table 3) | 1000-4000 rad/sec^2 (Fig 1). Worst case during gravity assisted shaking 8000-10000 rad/sec^2 for 16ms peak. 10216 rad/s^2 (max value), 8693 rad/s^2 (averaged over parameter combinations ) (table 3). | 61 (max value), 51 (averaged over different parameter combinations (table 3) | | | | | | $\Delta\omega - \alpha$ plots, scaled for brainmass. | Animal studies | Thibault and Margulies (1998), Duhaime et al (1987), Ommaya (1985) | Qualitative: one cannot conclude that shaking alone cannot cause fatal head injury. Critique on scaling injury thresholds. | Paper mentions occiput-back and chin-chest impacts and critsizes use of scaled animal tresholds. Paper suggest cumulative effect of repetitive sub-letal loading. |
| Duhaime, A. C. Gennarelli, T. A. Thibault, L. E. Bruce, D. A. Margulies, S. S. Wiser, R. | The shaken baby syndrome. A clinical, pathological, and biomechanical study | J Neurosurg 66 (1987) 409-15 | Test if infants particularly susceptible to injury due to shaking because of large head and weak neck make | Physical. | Head (diameter ~10 cm), neck single hinge, neck flexible link (length ~ 4cm), torso. Neck hinge-skull base = 3.3cm. | N/A | 1 mo | head 770-870g. Body 3-4 kg. | Human shaking | NA, both male and female. | Violent shaking in anterior-posterio direction. At least 20 trials per neck condition | | ~ 4 Hz (Fig. 1) | | 9.29 G tangential acceleration (mean of 69 trials) (Table 6). Tangential acceleration ranges between 5.70 G and 13.85 G dept on neck type (table 7) | | mean: 1138 rad/sec^2 at vertex, averaged over neck types and trials. (Table 6) | mean: 60.88 rad/s at vertex, averaged over neck types and trials. (Table 6) | | | | | | $\Delta\omega - \alpha$ plots, scaled for brainmass. | "scaled from primate experiments " not described how scaling took place. | Gennareli and Thibault [6] about duration and [16] Thibault and Gennarelli (1985) | Qualitative: IHI-ST is not usually caused by shaking alone. More likely that blunt impact plays a role | |
| Koizumi, T. Tsujiuchi, N. Hara, K. Miyazaki, Y. | Dynamic response and damage estimation of infant brain for vibration | 31st International Modal Analysis Conference on Structural Dynamics, IMAC (2013) 11-18 | study bridging vein stretch under various vibration frequencies | Physical, modified CRABI-6 | Skull, flexible neck, torso, single volume brain (silicon gel), cerebrospinal fluid, bridging veins (marker positions), Falx & tentorium. | Tables 2.2 - 2.5 | 6 mo | body 7.8kg (see crabi website http://www.humaneticsatd.com/crash-test-dummies/children/crabi-6mo) | Machine shaking | | | | 1.5 Hz, 2 Hz, 2.5 Hz, 3 Hz | 3 cm, 4 cm, 5 cm All combinations of freq and amplitudes tried. | | | | | | | | At freq = 3 Hz and amplitude > 4 cm, stretch ratio > 1.5. And 1.0-1.1 otherwise.(Fig 2.5 2.6) | | Bridging vein stretch > 1.5 | Cadaver studies and own FEM | [4] Lee, Haut (1988) | Qualitative: shaking frequency of 3 Hz is risk due to respons in bridging veins. Lower freqs (e.g. cradling) no risk. | |
| Lloyd, John Willey, Edward N. Galaznik, John G Lee, William E Luttner, Susan E | Biomechanical Evaluation of head kinematics during infant shaking versus pediatric activities of daily living. | Journal of forensic biomechanics 2 (2011) 1-9 | Compare infant head accelerations under normal vs. abusive situations | Physical. CRABI-12and NCSBS demonstration doll | Skull, neck multiple hinges, torso | | CRABI-12 mo, NCBS N/A | CRABI-12 body 9.97 kg, included head (see:http://www.humaneticsatd.com/crash-test-dummies/children/crabi-12m), NCBS 0.9 kg | Human shaking | 9 ( 7 male, 2 female), 20-77yo | Mild shaking, gravity assisted shaking, aggressive repetitive horizontal shaking. 3 times per doll and condition. 3 trials per doll | | 3-5 Hz | | 3.2-7.6 G (CRABI-12), 3.6-9.9 G (NCSBS-doll) | | 364-1068 rad/sec^2 (CRABI-12), 502-1587 rad/sec^2 (NCSBS-doll) at back of head | 12-25 rad/s (CRABI-12) 12-35 rad/s (NCSBS-doll) at back of head | | | | | | HIC-15 > 390. $\alpha$ > 10000 rad/sec^2 | Cadaver impact studies. Drop studies with CRABI-6. And own child experiments . | [13] De Preitere et al (2006) [39] Van Ee et al (2009) | Qualitative: shaking unlikely to be the primary cause of DAI. Values durinbg shaking are more or less the same for child in jumperoo | Shaking modes poorly explained. |

| General | | | | Model description | | | | | Model input | | | | | | Kinematic response | | | | Dynamic response | | | | | Injury criterium | | | Conclusions | Remarks |
|---|---|---|---|---|---|---|---|---|---|---|---|---|---|---|---|---|---|---|---|---|---|---|---|---|---|---|---|---|
| Authors | Title | Reference | Aim | Type of model | Modelled entities | Mat. props | Repr. Age | Repr. Weight | Type of shaking | Number of subjects shaking | Instructions to subjects | Duration of shaking | Frequency of shaking | Amplitude of shaking | (peak) linear acceleration | (peak) linear velocity | (peak) angular acceleration | (peak) angular velocity | skull | eye | brain | veins | neck | type of cryterium | source of tresholds | references | | |
| Miyazaki, Y. | The mechanism of shaken baby syndrome based on the visualization of intracranial brain motion | Japanese Journal of Neurosurgery 24 (2015) 468-476 | Visually determine bridging vein stretch during shaking | Physical. Modified CRABI-6 | Skull, torso, single volume brain, cerebrospinal fluid | Unable to determine from paper (Jananese) | | | Human shaking | | Shaking doll while keeping it upright ("standing"") and while the doll is sitting ("sitting"') (Fig 2) | | ~ 4 Hz (estimated from Fig. 4) | | | | | | | | | stretch ratio up to 4.0 - during shaking (Fig 4 ). Peak stretches of 5 and 3.5 for ""sitting" and "standing" shake. (Fig 7) | | stretch ratio in bridging veins | cadaver studies | [4] De Preitere et al. (2006) | (from abstract) relative displacements in violent shaking exceed thresholds for bridging vein rupture in most cases. Values were larger in shaking than in low height falls. Injury mechanism is reverse rotational motion between skull and brain due to change in rotation direction at endpoints. This is not present in falls | Paper is in Japanese. Informatin derived from abstract, Figures and their captions. |
| Prange, M. T.<br>Coats, B.<br>Duhaime, A. C.<br>Margulies, S. S. | Anthropomorphic simulations of falls, shakes, and inflicted impacts in infants | J Neurosurg 99 (2003) 143-50 | Compare rotational deceleration of head from different types of free falls to those during impact and shaking. | Physical. | Skull (diameter = 12.6 cm, weight = 1.13 kg), single hing neck (distance from COM head to centre of rotation = 9.2 cm, compares to C5-C6). | neck: heavy duty stainless steel hinge. Skull: polypropylene (American Platic, Fort Worth, TX; 2.25 mm thick) | 1.5 mo | body 4.83kg | Human shaking | 6 (4 male, 2 female) | Maximum effort, no release. 10 trials per subject. | > 5 cycles, last cycle ends with impact | ~2.3 Hz (Fig 2) | NA. Shake with largest amplitude is analysed | | | Peak values: Typical example: 2640 rad/sec^2 (Fig 2). Subject averaged data: ~4000 rad/sec^2 (Fig 5). Value derived from Fig 5 Measured at apex of head | Peak value: Typical example: 28 rad/s (Fig 2).No subject averaged data available. Measured at apex of head. | | | | | | $\Delta\omega - \alpha$ plots, scaled for brainmass. | Cadaver studies, primate experiments, angular velocities measuried in boxing | [1] Abel et al (1978), [15] Gennarelli et al (1982) [32] Margulies et al (1990), [39] Pincemaille et al (1988) | Qualitative: $\Delta\ \omega - \alpha$ are larger in impacts than is shaking. Falls and shakes have similar $\Delta\omega - \alpha$ . So chance of trauma is larger in falls. No threshold data $\Delta\ \omega - \alpha$ in shaking regime to support chance of trauma. | |
| Tomlinson, R. A.<br>Taylor, Z. A. | Photoelastic materials and methods for tissue biomechanics applications | Optical Engineering 54 (2015) 081208 | Use photoelastic material to visualize brain stresses. | Physical. 2D sagittal gelatine brain slice | single volume brain | Brain: gelatine / water in proportion 2:10, giving compressive modulus ~ 50 kPa. | N/A | N/A | Machine shaking | | | | | Max amplitude is 7.2 G | | | | | | | Max shear stress is 1150 Pa at brainstem (pg 081208-6) and 1180 (pg 081208-8) | | | treshold for permanent braindamage is 20 kPa (no ref given) | N/A | | Qualitative: results are in agreement with Duhaime: stresses in shaking are much smaller than threshold 20 kPa for damage. But state that model used is simple. | |
| Yamazaki, J.<br>Yoshida, M.<br>Mizunuma, H. | Experimental analyses of the retinal and subretinal haemorrhages accompanied by shaken baby syndrome/abusive head trauma using a dummy doll | Injury 45 (2014) 1196-206 | Measure pressure in eye of doll during shaking events. | Physical. Chou-chou baby doll with prop eye model. | Skull, single hing neck, torso, detailed eye anatomy. | Optimal percentage agar gel is 0.5% for infant vitreous body, based on surgical experience, giving G=0.7 kPa (Fig 5b). Experiments reported with 1% agar gel. | 1 mo | head/skull 800g. Mass of doll is 4 kg. | Human shaking on doll. Machine shaking on eye model. | 6 (5 female, 1 male) | Three shaking modes: 1. "fast". 2 "large amplitude". 3. "synchronized". Experments reported with freely chosen shaking mode and synchronized shaking (tables 2 & 3). | | 1.68 (synchronized) - 2.45 (no instruction to subject) (tables 2 & 3) | | averaged values of peak lin acceleration 20-60 m/s^2 at head of doll. (pg 1200). Freely chosen: 46 m/s^2 (averaged over subjects). Synchronized: 60 m/s^2 (averaged over subjects) (tables 2&3). | | | | | 0.85 kPa (compressive). 0.62 kPa (tensile) overaged over subjects (Tables 2 and 3) | | | | Timeintegral of stresses in eyeball, comparision of shaking and fall. | N/A | | Qualitative: mode of shaking is important for model response. Stresses proportional to accelerations. Timeintegral of eyeballstress in 1 cycle of shaking is larger than during fall. | |

| General | | | | Model description | | | | | Model input | | | | | | Kinematic response | | | | Dynamic response | | | | | Injury criterium | | | Conclusions | Remarks |
|---|---|---|---|---|---|---|---|---|---|---|---|---|---|---|---|---|---|---|---|---|---|---|---|---|---|---|---|---|
| Authors | Title | Reference | Aim | Type of model | Modelled entities | Mat. props | Repr. Age | Repr. Weight | Type of shaking | Number of subjects shaking | Instructions to subjects | Duration of shaking | Frequency of shaking | Amplitude of shaking | (peak) linear acceleration | (peak) linear velocity | (peak) angular acceleration | (peak) angular velocity | skull | eye | brain | veins | neck | type of cryterium | source of tresholds | references | | |
| Jenny, C. Shams, T Rangarajan, N, Fukuda, T. | Development of a biofidelic 2.5 kg infant dummy and its application to assessing infant head trauma during violent shaking | Proceedings of the 30th International Workshop on Human Subjects for Biomechanical Research (2002) | Develop better instrumented and realistic doll. | Physical. Aprica doll. Bodyweight = 2.5 kg. | Skull (weight = 772 gr, diameter = ~11 cm), single hinge neck (length = 53 mm), torso (weight = 1.2 kg). | | N/A | 10th percentile 2.5 kg japanese child. Skull/head 800g, torso 1.2kg. Data in table 1 specifies weights for single legs and arms. Then you get 2.52 kg. | Human | 1 Japanese male | | 4s | 4-5 Hz | | Max values: 27.7 G at head centre of gravity. 67.8 G at vertex. . Mean values: 26.2 G (centre of mass), 64.8 G (vertex). Averaged over 5 trials (table 2) | | 13.252 rad/sec^2 Max value for all trials. (page 139) | 153 rad/s Max value for all trials(page 139) | | | | | | | | | Only statement that kinematic parameters measured are larger than those measured by Duhaime. | |
| Jenny, C. A., Bertocci, G., Fukuda, T. ,Rangarajan, N., Shams, T. | Biomechanical Response of the Infant Head to Shaking: An Experimental Investigation | J Neurotrauma 34 (2017) 1579-1588 | Characterize biomechanical response of infant head during shaking using doll with improved biofidelity | Physical. Prop Aprica doll. | Skull (weight=772 g), neck (len gth=53 mm, weight=62 g), torso (weight=1244 g), arms, legs | | 5th percentile Jananese newborns | Bodyweight = 2.6 kg. | Human | 1 Japanes male | "violently shake dummy fore-aft for 3-4 sec" | 3-4 sec. 5 trials of at least 12 cycles/trial. | 4 Hz. | N /A | | | 7035 rad/s^2-10379 rad/sec^2 (Fig 4) Averaged within trials. Peak values for trials range between 9613-13260 rad/s^2 (Table 2) | 71.2 rad/s-98.4 rad/s (Fig 5) Averaged within trials. Peak values for trials range between 80-106 rad/s (table 2) | | | | | | Mentions injury threshold for concussion in primates and DAI in primates. Threshold concusion is exceeded, for DAI not in expts | primate studies | concussion: [31] Ommaya et al (2002) DAI: [29] Genanarelli et al (1982) | Predictions of risk based on publishec injury thresholds are not likely to be reliable given inherent limitations of these thresholds. Higher accelerations measured suggest higher potential for injury by shaking alone. | Appears to be an extended version of Jenny et al 2002. |

| General | | | | Model description | | | | | Model input | | | | | | Kinematic response | | | | Dynamic response | | | | | Injury criterium | | | Conclusions | Remarks |
|---|---|---|---|---|---|---|---|---|---|---|---|---|---|---|---|---|---|---|---|---|---|---|---|---|---|---|---|---|
| Authors | Title | Reference | Aim | Type of model | Modelled entities | Mat. props | Repr. Age | Repr. Weight | Type of shaking | Number of subjects shaking | Instructions to subjects | Duration of shaking | Frequency of shaking | Amplitude of shaking | (peak) linear acceleration | (peak) linear velocity | (peak) angular acceleration | (peak) angular velocity | skull | eye | brain | veins | neck | type of criterium | source of tresholds | references | | |
| Bandak, F. A. | Shaken baby syndrome: a biomechanics analysis of injury mechanisms | Forensic Sci Int 151 (2005) 71-9 | Injury biomechanics analysis of effects of reported angular velocities and accelerations in shaking for injury effect on head-neck | Mathematical | Skull (weight 0.68-1.59 kg), neck (length 3.81-6.35 cm). | | | | Mathematical. Single half shake. | | | | velocities assumed in Table 2. 50-150 rad/s and 5000-15000 rad/s^2 | | | 4.31 m/s (Table 2) | | | | | | | Neck distraction forces calculated to range between 1027-35931 N (table 3) | treshold for α (30000 rad/sec^2) and ω (50–120 rad/s). Tresholds on strength of infant neck. | previous research. Animal expts | [12] Duhaime et al (1987), [20] Nuckley et al (2000), [21] Ching et al (2001), [9] Duncan (1874) Mayer et al (1999) | Angular accelerations during shaking are too large to be supported by the neck. Criteria for SBS need to be revised. | Computed neck distraction forces are criticized in Margulies at al. Forensic science international 164 (2006) 268-269 as being 10 times too high |
| Batterbee, D. C. Sims, N. D. Becker, W. Worden, K. Rowson, J. | Computational model of an infant brain subjected to periodic motion simplified modelling and Bayesian sensitivity analysis | Proc Inst Mech Eng H 225 (2011) 1036-49 | Perform sensitivity analysis of model output (taken to be BV stretch) with respect to model parameters (e.g. material properties and geometry) (CSF thickness and viscosity and fontanel size, Brain E and E*, Gi and beta) | Mathematical. 2D FE model of sagital slice of head. | Skull, cerebrospinal fluid (5-8.6 mm thinkness), single volume brain, fontanelle (21-49 mm). See table 1. | Detailed props & geometry given. (table 1) | N/A | N/A | Mathematical. | | | | 4 Hz | (a) 93.18 mm sin translation at brainstem. (b) Idem + sine rotation of 30 deg amplitude. Amplitude acceleration = 3G. | | | | | | | | Stretch ratrio of bridging veins ~0.2 for sine translation (Fig 4). Ratio = 2 for sine translation + rotation. Both dept on parameter choices. Data reported appears to be strain instead of stretch ratio, given their explanation in Conclusion section (pg 1047) | | | | | Sensitivity of the model outputs to parameters values depends on the shaking conditions. Particularly, density ratio, CSF thickness and fontanelle size have sensitivity that depends on excitation type because they affect buoyance effects, which are more dominant in translational than in rotational excitation.Buoyance effects damp brain motion in translational excit. Less in translational and rotational. | |
| Batterbee, D. C. Sims, N. D. Rowson, J. | Finite element modelling of shaken baby syndrome: A frequency response approach | 27th Conference and Exposition on Structural Dynamics (2009) IMAC XXVII | Develop simplified FE infant head model for shaking. Investigate influence of fontanelle | Mathematical. 2D FE model of sagital slice of head. | Skull (diameter = 100 mm), single volume brain (diameter= 80 mm), cerebrospinal fluid, fontanelle (size = 40 mm). | E, rho, Ey and nu given for all materials. Based on "readily available materials with biological like properties" (table 2) | N/A | N/A | Mathematical. | | | 5 cycles | 2-20 Hz | sine acceleration with amplitude of 29.4 m/sec^2. | | | | | | | Stresses are larger near fontanelle (Fig 9). In model without fontanelle largest stresses at brainstem, but very small. | | | | | | Fontanelle reduces buoyance effect and increases likelyhood of bridging vein tears. Larger stresses at top of brain might lead to damage. Injury criterium likely different for fused and unfused skulls. | |
| Bondy, M. Altenhof, W. Chen, X. Snowdon, A. Vrkljan, B. | Development of a finite element/multi-body model of a newborn infant for restraint analysis and design | Comput Methods Biomech Biomed Engin 17 (2014) 149-62 | Model validation. | Mathematical. 3D 17 segment RBM / FE model, based on Nita newborn demonstration doll | Head, 7 segment neck, torso, upper and lower limbs. | Scaled from adult. | Nita doll matches 32-33 wks gestational age newborn | body 1.9 kg, head 0.528 kg | Mathematical, based on human shaking datafrom Wolfson et al (see below) | | | | ~4 Hz (fig 7) | Accelaration applied to torso with max amplitude ~80 m/s^2 (Fig 7) | | | 18567-21205 rad/sec^2, dept on stiffness parameters (table 3) | 39-45 rad/s, dept on stiffness parameters (table 3). | | | | | | Δmax ω – αmax plots (Fig 8) | Primate studies, privious doll studies | Ommaya (1985), Duhaime et al (1987), Cory and Jones (2003) | Results are in line with previous studies | |

| General | | | | Model description | | | | | Model input | | | | | | Kinematic response | | | | Dynamic response | | | | | Injury criterium | | | Conclusions | Remarks |
|---|---|---|---|---|---|---|---|---|---|---|---|---|---|---|---|---|---|---|---|---|---|---|---|---|---|---|---|---|
| Authors | Title | Reference | Aim | Type of model | Modelled entities | Mat. props | Repr. Age | Repr. Weight | Type of shaking | Number of subjects shaking | Instructions to subjects | Duration of shaking | Frequency of shaking | Amplitude of shaking | (peak) linear acceleration | (peak) linear velocity | (peak) angular acceleration | (peak) angular velocity | skull | eye | brain | veins | neck | type of criterium | source of tresholds | references | | |
| Cheng, J. Batterbee, D. Yoxall, A. Sims, N. D. Rowson, J. Howard, I. C. | Shaken baby syndrome: A structural dynamics perspective | 23rd International Conference on Noise and Vibration Engineering (2008) 2003-2014 | Use numerically modelling to investigate suggested key role of fontanelle in IHI-ST | Mathematical. 2D FE model of sagittal slice of head and 3D model. | Skull , single volume brain, cerebrospinal fluid, fontanelle | Values given in Tables 1 and 2. Values in Table 1 chosen to match experimental facility, rather then biological specimens. No specification for Table 2. | N/A | N/A | Mathematical | | | 2 cycles | 4 Hz | sin excitation in lateral direction, max amplitude = 1.2 m/s. | | | | | | | highest stresses in fontanell area ~1 MPa (Fig 5). Displacement at top of brain ~ .6 mm (Fig 5) | | | | | | Qualitative: presence of fontanelle could lead to greater chance of SDH due to shaking. | |
| Cheng, J. Howard, I. C. Rennison, M. | Study of an infant brain subjected to periodic motion via a custom experimental apparatus design and finite element modelling | J Biomech 43 (2010) 2887-96 | Develop FE model for IHI-ST and compare different modeling techniques for brain-skull interface | Mathematical. 2D FE model of sagittal slice of head. | Skull, single volume brain, cerebrospinal fluid, fontanelle. | Given in Table 3. Values derived for mechanical surrogate developed in paper. | N/A | N/A | Mathermatical, using input from experimental model (Fig 9). | | | 4-7s, but 3.5s in fig | ~ 2-3 Hz (Fig 9, 13-14) | | | | | | | | | Displacement of markers 2-6 mmm, dept on modelling method (Fig 13, 15). | | | | | Special features of infant skulls, such as fontanelle, are fundamentally important to understand how the head behaves when shaken. | Technical comments on FE modelling technique of CFS. Hint at resonant build-up of brain motion near fontanelle. |
| Cirovic, S. Bhola, R. M. Hose, D. R. Howard, I. C. Lawford, P. V. Parsons, M. A. | Mechanistic hypothesis for eye injury in infant shaking : An experimental and computational study | Forensic Sci Med Pathol 1 (2005) 53-9 | Determine pressure on eye | Mathematical. FE model of rabbit eye. | Detailed eye anatomy (eye (diameter = 24 mm), vitreous, orbit, orbital bone, fa, sclera, eye muscles) | Material props of fat, vitreous, sclera taken from literature (Power et al 2002), Parameters for ocular muscles from literature (Robinson) | N/A | N/A | Mathematical | | | | 200 Hz sine wave.Chosen to be equal to resonance freq of modeled system. | displacement of max amplitude 0.1 mm applied to orbital bone | | | | | | max displacement is 0.8 mm at centre of eye. (Fig 5). Max stress = ~12 MPa at orbit (Fig 6) | | | | | | | Qualitative: resonance effects may lead to buildup of displacement and stresses during shaking. | |
| Coats, B. Eucker, S. A. Sullivan, S. Margulies, S. S. | Finite element model predictions of intracranial hemorrhage from non-impact, rapid head rotations in the piglet | Int J Dev Neurosci 30 (2012) 191-200 | Aim1: Find best model type related to strain and displacement of brain. Aim2: best predictor parameters for piglet intercranial hemorrhage (IH). | Mathematical. 3D FE model of piglet brain. | skull, brain, brainstem, falx, CSF, Two models for pia-arachnoid complex: spring connector and solid element | Table 1. Animal tests literature. But results only matched when these were taken as 1.5 or 2.25 times the values. | 3-5 day old piglets | Brain 33-39.5g | Uses machine based axial, sagittal and coronal head rotations from animal experiments (Ibrahim 2010) for model validation | | | | | | | | In animal expts: 26.000 - 85000 rad/s^2 (pg 194) | In animal experiments: 130 - 220 rad/s (pg 194) | | | Aim 1: brain shear modulus for model of brain in situ needed to be adjusted by 1.5-2.25 times as compared to expt values for brain in vivo to get best match with data from expts on 3-5 day old pigs | Aim 2: by numerical techniques identified beste predictor for IH and corrsponding threshold value for max strain | | Max strain in spring connectors and % connectors which have this strain during simulations. Max strain = 31% best matches experimental data. For solid element model: 45.4 kPa stress on 10% of outer surface | Numerical optimization by comparing IH under various expt conditions to strain (spring connector model) and stress (solid element model) in corresponding model simulations for different threshold values | | Sagittal rotations give most damage in animal expts. Best predictor for IH in 3-5 day old piglets is FE model with spring connectors for pia-arachnoid complex . Peak strain for 1% of connectors best predicts IH. Best threshold for IH prediction is 31% strain. | |

| General | | | | Model description | | | | | Model input | | | | | | Kinematic response | | | | Dynamic response | | | | | Injury criterium | | | Conclusions | Remarks |
|---|---|---|---|---|---|---|---|---|---|---|---|---|---|---|---|---|---|---|---|---|---|---|---|---|---|---|---|---|
| Authors | Title | Reference | Aim | Type of model | Modelled entities | Mat. props | Repr. Age | Repr. Weight | Type of shaking | Number of subjects shaking | Instructions to subjects | Duration of shaking | Frequency of shaking | Amplitude of shaking | (peak) linear acceleration | (peak) linear velocity | (peak) angular acceleration | (peak) angular velocity | skull | eye | brain | veins | neck | type of criterium | source of tresholds | references | | |
| Couper, Z. Albermani, F. | Infant brain subjected to oscillatory loading: material differentiation, properties, and interface conditions | Biomech Model Mechanobiol 7 (2008) 105-25 | 1) Compare effects of modelling strategies and material properties. 2) Find qualitative effects on brain stress and strain and CSF pressures. | Mathematical: FE model of 2D axial slice of head of child of 3 months old. | Skull, single volume brain, cerebrospinal fluid. | Mentioned in Table 1 and Fig 2. Data taken from literature. Different modelling strategies and parameter values are shown in Table 2 and their effect is investigated | 3 mo | N/A | Mathematical | | | 2s (8 cycles) | 4 Hz | Sine shaped acceleration in anterior-posterior direction, amplitude = 60 m/sec^2. | | | | | | | Displacements at corpus callosum ~ 1-2 mm dept on modelling condition. Pressure in CSF. (Figs 11-15) | | | | | | Qualitative: brain-CSF interaction depts on volume subarachnoid space andthickness variations associated with giri. Differating between types of brainmatterr changes evulation of brain-CSF interaction. | |
| Couper, Z. Albermani, F. | Mechanical response of infant brain to manually inflicted shaking | Proc Inst Mech Eng H 224 (2010) 1-15 | Numerical modeling of the content of an infant head during shaking to provide improved insight into associated mechanics and resulting head trauma | Mathmatical. 3D FE model of brain of 3 mounths old child. CSF not as solid but using fludi dynamics. | Skull, single volume brain, cerebrospinal fluid | Not explicitely mentioned. Reference to previous work by same authors and literature | 3 mo | N/A | Mathematical shaking, based on kinematics of surrogate experiments in other papers of Couper and Albermani. Detailed in Fig 4. | | | 0.25 s due to computational complexity. | 4 Hz | | Y: -100 - 50 m/s^2. Z: -30 - 100 m/s^2.(Fig 4) | | 1000 - 500 rad /sec^2 (Fig 4) | 20 - 35 rad/s (Fig 4) | | | (peak) stresses and strains in corpus callosum and posterior aspect (Fig 10 + text on page 12) | Peak bridging vein stretch ratios upto 2.15 for some area's (Fig 11) | | thresholds in bridging vein stretch > 1.5. Brain tissue strains > 0.1 (axonal injury) | cadaver studies, animal expts | [31] Lee and Haut (1989). [13] Morrison et al (2003) | Qualitative: strains in shaking simulations increase likelihood of focal axonal injury at contact locations and deep brain structures and have a capacity to lead to SDH due to bridging vein rupture. | |
| Couper, Z. S. Albermani, F. | Infant brain subjected to oscillatory loading | Australian Journal of Mechanical Engineering 6 (2007) 79-85 | Develop 2D FE model of brain with novel method for CFS modeling | Mathematica. FE model of 2D axial slice of head | | Literature values, "in alignement with other researchers". | 3 mo | N/A | Mathematical | | | 2 s (8 cycles) | 4 Hz sine wave | Acceleration amplitude = 60 m/sec^2 in sagittal plane | | | | | | | displacement at corpus callosum ~ 1-3 mm, dept on subarachnoid space volume (Fig 2) | | | | | | Qualitative: displacement and strains in brain highly dependent on volume of subarachmnoid space. Higher volume leads to decrease of stress. Also protrusion of giri important | |
| Hans, S. A. Bawab, S. Y. Woodhouse, M. L. | A finite element infant eye model to investigate retinal forces in shaken baby syndrome | Graefes Arch Clin Exp Ophthalmol 247 (2009) 561-71 | Develop FE model to assess forces at retina in shaking and impact motions | Mathematical. FE model of eye, head and neck | Skull, neck single hinge (length = 4cm), single volume brain, detailed eye anatomy (cornea, sclera, vitreous, aqueous, ciliary body, lens, retina, choroid, fat, tendons, eye muscles) | Mentioned in Tables 1 and 2. Based on literature references, some of which are other modelling | N/A | Skull 820 g, brain 500g, based on Duhaime et al. | Mathematical | | | 1 s | 4 Hz sine wave | displacement about neck pivot with max amplitude of 60 degrees | | | | | | mean retinal nodal forces are 0.05-0.08 N during shaking. Peaks up to 0.45 N (Fig 10) | | | | threshold for retinal adhesive force is 0.14 N for monkey eyes | primate experiments | [49] Kita and Marmor (1992), [50] Kita and Marmor (1992). | Qualitative: forces on retina are larger during shaking than in impact. And may cause retinal hemorrhaging. Repetitive shaking builds up forces on retina. | |

| General | | | | Model description | | | | | Model input | | | | | | Kinematic response | | | | Dynamic response | | | | | Injury criterium | | | Conclusions | Remarks |
|---|---|---|---|---|---|---|---|---|---|---|---|---|---|---|---|---|---|---|---|---|---|---|---|---|---|---|---|---|
| Authors | Title | Reference | Aim | Type of model | Modelled entities | Mat. props | Repr. Age | Repr. Weight | Type of shaking | Number of subjects shaking | Instructions to subjects | Duration of shaking | Frequency of shaking | Amplitude of shaking | (peak) linear acceleration | (peak) linear velocity | (peak) angular acceleration | (peak) angular velocity | skull | eye | brain | veins | neck | type of criterium | source of tresholds | references | | |
| Jones, M. D. Martin, P. S. Williams, J. M. Kemp, A. M. Theobald, P. | Development of a computational biomechanical infant model for the investigation of infant head injury by shaking | Medicine, Science and the Law 55 (2014) 291-299 | Find effect of quasi-static and rate-dependent neck stiffness on head linear acceleration and rotational max acceleration and speed. | Mathematical. 3D RBM | Head (diameter ~ 6.6 cm), multiple hinge neck, torso, lims, spine. | Mechanical parameters from literature and MRIs. Some stiffness and damping parameters determined by numerical optimization. | 9 mo | Head 2.3kg | Mathematical. | | | | 3 Hz sine wave | max amplitude is 65 mm at torso in anterior posterior direction | 95.73 m/s^2 Ranges between 80-350 when joint stiffness is varied | | 1133 rad/s^2 | 17.17 rad/s | | | | | | $\Delta\omega-\alpha$ plots (Fig 6). Note : Fig 6 is wrong and contains information of Fig 5b. | animal expt + previous (doll) studies. | [11] Cory and Jones (2003), [12] Duhaime et al (1987), [32] Ommaya (1985), [33] Klinich et al (1996), [34] Thibault and Margulies (1998). | Model kinematics comparable to doll studies. Neck stiffness properties are important for peak vertex acceleration. Accelerations below injury thresholds from literature. Doubt on validity of scaling tresholds from animal expts. | **Paper contains a wrong figure 6.** |
| Lintern, T. O. Puhulwelle Gamage, N. T. Bloomfield, F. H. Kelly, P. Finch, M. C. Taberner, A. J. Nash, M. P. Nielsen, P. M. F. | Head kinematics during shaking associated with abusive head trauma | Journal of Biomechanics 48 (2015) 3123-3127 | Validate developed coupled rigid body computational model for lamb to reproduce in vivo lamb shaking | Mathematical. 2D RBM of lamb. | Skull, multiple hinge neck, torso. | | Lambs 5-8 days | Lambs 7-8.8kg | Torso kinematics measured during shaking lamb experiments | | | | ~2 Hz (from Fig 2). | | 100-200 m/s^2 after optimization of model parameters. (Fig 4) Text on pg 3125 states 200-250 m/s^2. | | | ~20 rad/s after optimization (Fig 4) | | | | | | | | | Head kinematics during shaking can be reproduced by a RBM and can discribe head-torso impacts. | |
| Morison, Christopher Neil | The dynamics of shaken baby syndrome | PhD thesis Univeristy of Birmingham (2002) | creating 3D FEM for IHI-ST including CSF. Finding BV stresses and strains during shaking | Mathematical. 3D FE model | Skull, single volume brain, cerebrospinal fluid, spine, tentorium. | Partly from literature, partly from own data. See chapter 5 | 11 wks | N/A | Mathermatical. Angular displacement imposed on skull. | | | | 4Hz | | 60 degrees amplitude (Fig 5.7) | | | | | | Stresses increase from brainstem to vertex (Fig 5.20). Max value is 800 Pa. | bridging veins stretch ratio between 0.8 and 1.25 (Fig 5.8). | | bridging vein stretch ratio | literature and own independent experiments | Lowenhielm (1974), Lee and Haut (1989), Meaney (1991) | Rotational component of shaking responsible for 93% of BV stretch. Bridging veins tresholds of 1.5 might be too large for children. | Contains possible more interesting data. Do not know why it never is published as a paper. Looks like solid work. |
| Nadarasa, J. Deck, C. Meyer, F. Raul, J. S. Willinger, R. | Infant eye finite element model to investigate retinal hemorrhages after fall and shaking events | Comput Methods Biomech Biomed Engin 18 (2015) 2016-7 | Compare retinal hemorrhage between domestic falling and shaking. | Mathematical. FE eye model. | Detailed eye anatomy (sklera,choroid, retina, vitreous, zonules, lens, ciliary body, aqueous, cornea, extra-ocular muscles, optic nerve, fromt membrane, orbital fat, orbital wall). | statement that mechanical properties were taken fro m literature and MRI, no refs given. | 6 wk for Q0 dummy | 3.46 kg for Q0 dummy | Kinematics of shaking Q0 dummy (body mass = 3.46 kg, length = 59.7 cm, age = 6 months) is used as input for the eye model. | | | 200 ms (1 shake period) | 5 Hz | | 9-12 G when shaking Q0-doll (Table 1) | | 2358-4961 rad/sec^2 when shaking doll (Table 1) | | | Pressure: 1.5-2 kPa at posterior pole, extending to mid-retina | | | | | | | Retinal haemmorrhages are more likely due to rotational accelerations than to pure linear ones. Shaking is more dangerous than domestic falls for retinal hemorrhage.Pressure in eye 4 x and Mises strain 14 x higher in shaking than in falls | |

| General | | | | Model description | | | | | Model input | | | | | | Kinematic response | | | | Dynamic response | | | | | Injury criterium | | | Conclusions | Remarks |
|---|---|---|---|---|---|---|---|---|---|---|---|---|---|---|---|---|---|---|---|---|---|---|---|---|---|---|---|---|
| Authors | Title | Reference | Aim | Type of model | Modelled entities | Mat. props | Repr. Age | Repr. Weight | Type of shaking | Number of subjects shaking | Instructions to subjects | Duration of shaking | Frequency of shaking | Amplitude of shaking | (peak) linear acceleration | (peak) linear velocity | (peak) angular acceleration | (peak) angular velocity | skull | eye | brain | veins | neck | type of criterium | source of tresholds | references | | |
| Ponce, E.<br>Ponce, D. | Modeling neck and brain injuries in infants | IEEE Comput Graph Appl 31 (2011) 90-6 | Predict effect of shaking on vertebrae C1-C4 and diffuse alterations in brain | Mathematical. 3D FE model | Skull, single volume brain, cerebrospinal fluid, spinal cord. | Literature values (table 1) from two other modelling studies | 6-9 mo (page 92) | N/A | | | | | 3 Hz sine | 200 N transverse force at C4. | | | | | | | Central brain regions show largest displacements (Figs 1-6). Stresses are more or less uniformly distributed. | | | Von Mises stress > 0.048 N/mm^2 = 50 % chance on injury. Stress > 0.08 N/mm^2 = 100 % chance on injury. | | [5] Meyer et al (2010) | | FEM appears to be a practical, universal, economical and fast tool with important forensic use. |
| Rangarajan, N.<br>Kamalakkannan, S. B.<br>Hasija, V.<br>Shams, T.<br>Jenny, C.<br>Serbanescu, I.<br>Ho, J.<br>Rusinek, M.<br>Levin, A. V. | Finite element model of ocular injury in abusive head trauma | J aapos 13 (2009) 364-9 | Create model for simulating force and deformation effects on eye during shaking. | Mathematical. FE eye model. | Detailed eye anatomy (orbit [32x32x50mm], fat, sclera [diam=20mm, thickness=1mm], retina [diam=18mm, thickness=0.25 mm], vitreous [diam=18mm]). Muscle modelled as spring/dampers | From literature. Details are in e-supplement 2,3,4. Sclera & retina: Young modulus=3.58 MPa. Vitreous: viscoelastic (K=0.7 or 7.0 MPa) or Fluid (VC=0.1-0.5 Pa/mm.s) Fat: elastic (E=0.047 MPa) or viscoelastic (K=0.7 MPa) | full term newborn 4-7mo (for some parameters) | N/A . Input to drive model based on shaking 2.5 kg dummy | Mathematical | | | 1 pulse, based on shaking 2.5 kg dummy | 5 Hz | 50 rad/s about orbit. Linearly increasing and descreasing velocity profile with peak at 50 rad/s (suppl 6) | | | | | | stresses 14-120 kPa, dept on modelling details. Largest stresses at ora serrata, which is interface between retina and vitreous.(Fig 2) | | | | Locations of retinal bleeding from case literature. | | | Qualitative: area with largest stresses coincides with location where haemorrhages are observed: junction of retina and vitreous posterior pole. There is build-up effect of stress during multiple shakes. First shake gives much lower stress and strain. | |
| Raul, J. S.<br>Roth, S.<br>Ludes, B.<br>Willinger, R. | Influence of the benign enlargement of the subarachnoid space on the bridging veins strain during a shaking event: a finite element study | Int J Legal Med 122 (2008) 337-40 | Effect of size of subarachnoid space on BV stretching during shaking | Mathematical. 3D FE model | Skull (circumference = 45-55 cm), single volume brain, cerebrospinal fluid. Width of subarachnoid space is varied between 2mm (standard) up to 8 mm (BESS). | Literature. Refers to paper by Roth [12] (see below) | 6 mo | N/A | Mathematical, based on exp data from Prange. | | | 1 cycle of 400 ms | 2.5 Hz sinewave | sine velocity pattern with amplitude 30 rad/s, applied at C5-C6. | | | | | | | | Peak strain in bridging veins decrease with increases size of subaranoid space.At vertex peak strain is 0.9 for 2mm and 0.22 for 8 mm. (Fig 2) Corresponding stretch ration's are 1.9 (2mm) and 1.22 (8mm). In occipital area stretch ratios 1.55 (2mm) and 1.34 (8mm) | | Weakest point of BV is in subdural and not subarachnoid | [19] | | Increased size of subarachnoid space does not lead to increased risk of subdural bleeding due to increased damping effect of CSF. | |

| General | | | | Model description | | | | | Model input | | | | | | Kinematic response | | | | Dynamic response | | | | | Injury criterium | | | Conclusions | Remarks |
|---|---|---|---|---|---|---|---|---|---|---|---|---|---|---|---|---|---|---|---|---|---|---|---|---|---|---|---|---|
| Authors | Title | Reference | Aim | Type of model | Modelled entities | Mat. props | Repr. Age | Repr. Weight | Type of shaking | Number of subjects shaking | Instructions to subjects | Duration of shaking | Frequency of shaking | Amplitude of shaking | (peak) linear acceleration | (peak) linear velocity | (peak) angular acceleration | (peak) angular velocity | skull | eye | brain | veins | neck | type of criterium | source of tresholds | references | | |
| Roth, S. Raul, J. S. Ludes, B. Willinger, R. | Finite element analysis of impact and shaking inflicted to a child | Int J Legal Med 121 (2007) 223-8 | Compare intracerebral dynamic response in impact and shake using new FEM model. | Mathematical. 3D FE model o | Skull, single volume brain, cerebrospinal fluid, bridging veins, fontanelle | All literature: some child [9,10], most pig or adult. Table 1. | 6 mo | N/A | Mathematical, based on exp. Data from Prange et al. | | | 1 cycle of 400 ms | 2.5 Hz sinewave | sine velocity pattern with amplitude 30 rad/s, applied at C5-C6. | | | | | | | Max Von Mises stress is 3.2 kPa at vertex. Max pressure is 22 kPa at frontal area | Max bridging veins strain is 90 %. Stretch ratio = 1.9. | | bridging veins stretch | cadaver studies | [16] Lee, Haut | Bridging vein stretch in shaking and impact are in regime to be able to cause rupture. For SDH, model shows that shaking can haved same consequences as impact, but brain pressure is less in shaking. | Critique on use of kinematic parameters to predict brain injury. |
| Wolfson, D. R. McNally, D. S. Clifford, M. J. Vloeberghs, M. | Rigid-body modelling of shaken baby syndrome | Proc Inst Mech Eng H 219 (2005) 63-70 | Investigate effect of neck stiffness on head motion and head-torso impacts as injury mechanism. | Mathematical. 3D RBM adapted from MADYMO CRABI-1year model. | Skull, single hinge neck, torso. In head-torso impact expts two-hinge neck. | | 1 yr | N/A. Internet information suggests about 10 kg. | Mathematical, using as input acceleration data from human shaking experiments on a 6 mo prop doll. | 10 (doll expt) | "as long and hard as possible" (doll expt) | max 22 s (mean 11.2 s) (doll expt) | max 5.5 Hz (mean 3.5 Hz) (doll expt) | peak toro acceleration 6-8 G (Fig 1). RMS value = 3 G. (doll expt) | 30 m/s^2 in accompanying doll expt | | < 1000 rad/s^2 (Fig 4). Pg 65 mentions outlier at 14000 rad/s^2 due to meeting end-stop constraint. In head-torso impact study: max ~ 10.000 rad/s^2 (fig 5) | ~ 20 rad/s (Fig 4) Text (pg 65) mentions 20 rad/s + outlier at 130 rad/s due to meeting end-stop constraint. In head-torso mpact: max ~95 rad/s (fig 5) | | | | | | $\Delta\omega - \alpha$ plots (Fig 4) | primate expt and previous studies (not specified in text, but presumably the references given in next column) | [15] Duhaime et al (1987), [16] Cory and Jones (2003), [24] Thibault and Margulies (1998), [20] Ommaya (1995), [23] Klinich et al (1996) . | impact-type charateristics are required to exceed current injury criteria. In impacts only lower values for injury threshold were exceeded. Research on tissue props more important to understand IHI-ST. | Paper contains critique on using scaled, impact based tresholds as injury criterium. Suggestion there might be effect of cumulative loading. |
| Yoshida, M. Yamazaki, J. Mizunuma, H. | A finite element analysis of the retinal hemorrhages accompanied by shaken baby syndrome/abusive head trauma | J Biomech 47 (2014) 3454-8 | Analyse effect of shaking on stress tensors in retina using FEM . | Mathematical. FE eye model. | Detailed eye anatomy (orbit d=16mm, fat, sclera, retina, vitreous, cornea). Eye diameter = 10mm | Literature, various ages | NA. Paper is follow-up of paper by Yamazaki on mechanical model of 1 mo child. | N/A | Mathematical | | | | 2.5 Hz sinewave | 30 cm in sagittal plane | 40 m/sec^2 (Fig 3) | | | | | normal component stress = 1000 Pa. Shear component = 3 Pa (Fig 4). Location of max stress is posterior pole. | | | | | | | timeintegral of stress is indicator of retina haemmorhage. Timeintegral larger in one cycle of shaking than in single impact. Supports build-up effect. | |
